# Collective behavior from surprise minimization

**DOI:** 10.1073/pnas.2320239121

**Published:** 2024-04-17

**Authors:** Conor Heins, Beren Millidge, Lancelot Da Costa, Richard P. Mann, Karl J. Friston, Iain D. Couzin

**Affiliations:** ^a^Department of Collective Behaviour, Max Planck Institute of Animal Behavior, KonstanzD-78457, Germany; ^b^Centre for the Advanced Study of Collective Behaviour, University of Konstanz, KonstanzD-78457, Germany; ^c^Department of Biology, University of Konstanz, KonstanzD-78457, Germany; ^d^VERSES Research Lab, Los Angeles, CA90016; ^e^Medical Research Council Brain Networks Dynamics Unit, University of Oxford, OxfordOX1 3TH, United Kingdom; ^f^Department of Mathematics, Imperial College London, LondonSW7 2AZ, United Kingdom; ^g^Wellcome Centre for Human Neuroimaging, University College London, LondonWC1N 3AR, United Kingdom; ^h^Department of Statistics, School of Mathematics, University of Leeds, LeedsLS2 9JT, United Kingdom

**Keywords:** collective motion, active inference, agent-based models, Bayesian inference, animal behavior

## Abstract

Collective motion is ubiquitous in nature; groups of animals, such as fish, birds, and ungulates appear to move as a whole, exhibiting a rich behavioral repertoire that ranges from directed movement to milling to disordered swarming. Typically, such macroscopic patterns arise from decentralized, local interactions among constituent components (e.g., individual fish in a school). Preeminent models of this process describe individuals as self-propelled particles, subject to self-generated motion and “social forces” such as short-range repulsion and long-range attraction or alignment. However, organisms are not particles; they are probabilistic decision-makers. Here, we introduce an approach to modeling collective behavior based on active inference. This cognitive framework casts behavior as the consequence of a single imperative: to minimize surprise. We demonstrate that many empirically observed collective phenomena, including cohesion, milling, and directed motion, emerge naturally when considering behavior as driven by active Bayesian inference—without explicitly building behavioral rules or goals into individual agents. Furthermore, we show that active inference can recover and generalize the classical notion of social forces as agents attempt to suppress prediction errors that conflict with their expectations. By exploring the parameter space of the belief-based model, we reveal nontrivial relationships between the individual beliefs and group properties like polarization and the tendency to visit different collective states. We also explore how individual beliefs about uncertainty determine collective decision-making accuracy. Finally, we show how agents can update their generative model over time, resulting in groups that are collectively more sensitive to external fluctuations and encode information more robustly.

## Significance

We introduce a model of collective behavior, proposing that individual members within a group, such as a school of fish or a flock of birds, act to minimize surprise. This active inference approach naturally generates well-known collective phenomena such as cohesion and directed movement without explicit behavioral rules. Our model reveals intricate relationships between individual beliefs and group properties, demonstrating that beliefs about uncertainty can shape collective decision-making accuracy. As agents update their generative model in real time, groups become more sensitive to external perturbations and more robust in encoding information. Our work provides fresh insights into understanding collective dynamics and could inspire strategies in the study of animal behavior, swarm robotics, and distributed systems.

The principles underlying coordinated group behaviors in animals have inspired research in disciplines ranging from zoology to engineering to physics (1–3). Collective motion in particular has been a popular phenomenon to study, due in part to its striking visual manifestation and ubiquity (e.g., swarming locusts, schooling fish, flocking birds, and herding ungulates), and in part to the simplicity of models that can reproduce many of its qualitative features; like cohesive, directed movement (4–7). Because of this, collective motion is often cited as a canonical example of a self-organizing complex system, wherein collective properties emerge from simple interactions among distributed components.

Popular theoretical models cast collective motion as groups composed of self-propelled particles (SPPs) that influence one another via simple “social forces.” Early models like the Vicsek model (6) consider only a simple alignment interaction, where each particle aligns its direction of travel with the average heading of its neighbors. While oversimplifying the biological mechanisms in play, SPP models—like the Vicsek model—are useful for their amenability to formal understanding, e.g., the computation of universal quantities and relations through hydrodynamic and mean-field limits (8–11).

Recent research has shifted toward more biologically motivated approaches that aim to model the specific behavioral circuits and decision-rules that govern individual behaviors (12–15). While these models are less analytically tractable than SPP models, they are more appealing to domain specialists like biologists, as they can generate predictions about sensory features in an individual’s environment that are necessary and sufficient for evoking behavior. Furthermore, these predictions can be tested experimentally (14, 16). This data-driven approach can thus provide mechanistic insights into the biological and cognitive origins of decision-making (13, 17).

In this work, we propose a model class that blends the first-principles, theoretical approach of physical models with biological plausibility, resulting in an ecologically valid but theoretically grounded agent-based model of collective behavior. Our model class is based on active inference, a framework for designing and describing adaptive systems where all aspects of cognition—learning, planning, perception, and action—are viewed as a process of inference (18–21). Active inference originated in theoretical neuroscience as a normative account of self-organizing, biological systems as constantly engaged in predictive exchanges with their sensory environments (22–25).

## Collective Motion Models: From SPP to Bayesian Agents

In popular SPP models, an individual’s movement is described as driven by a combination of social and environmental forces. These forces are often treated as vectors that capture various tendencies seen in biological collective motion, such as repulsion, attraction (to neighbors or external targets), and alignment. These forces can then be combined with various nonlinearities and weights to capture mechanisms of interaction.

**Fig. 1.**
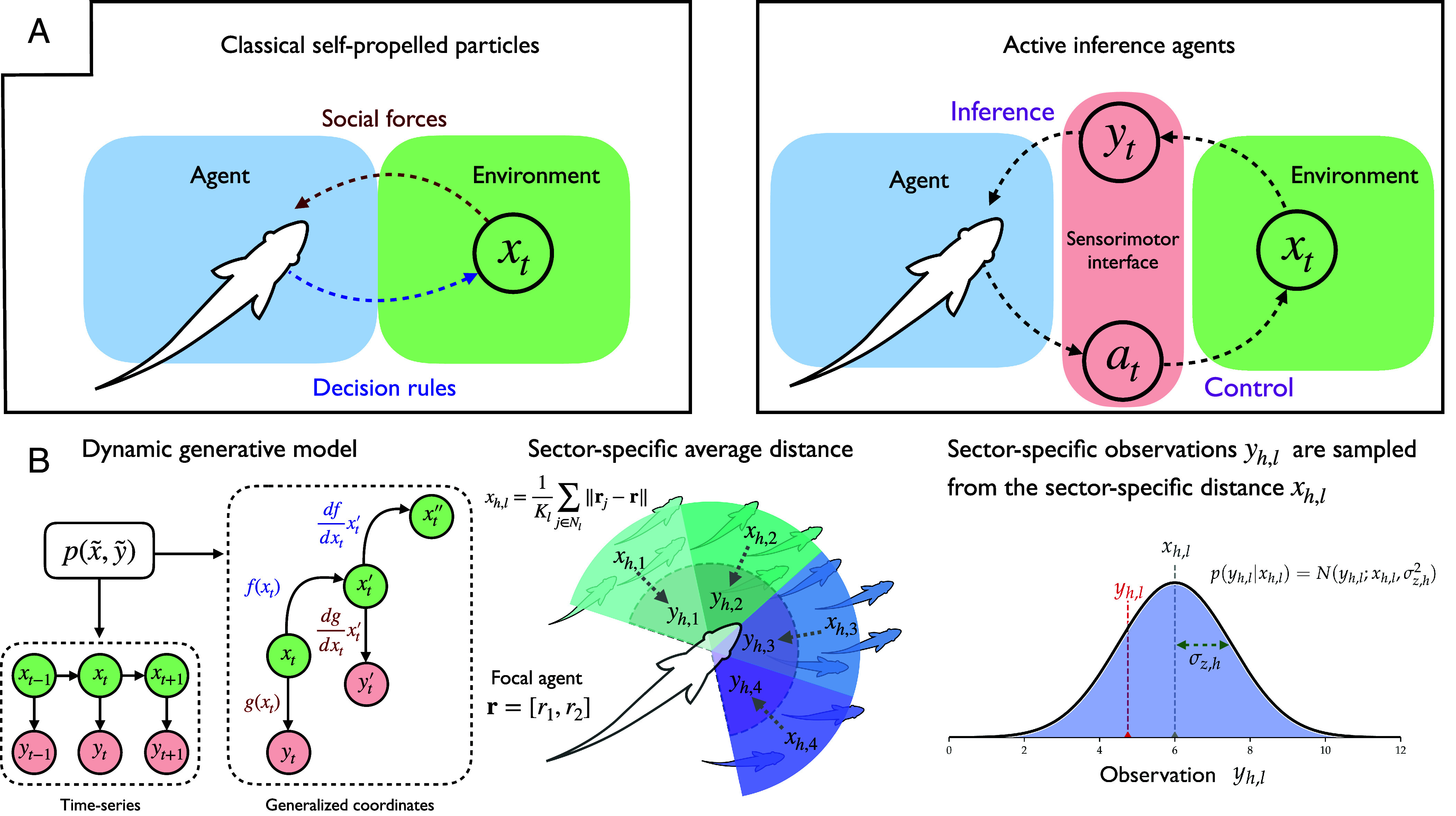
(*A*) Schematic illustrating the Bayesian perspective in the context of our single agents, where the hidden states of the environment are segregated from a focal agent by means of sensory data $y_{t}$ (*Right* panel of *A*). This contrasts with classic SPP models (*Left* panel of *A*), where environmental or social information manifests in terms of social forces on the focal individual, who emits its own actions based on hand-crafted decision-rules (e.g., changes to heading direction). (*B*) Schematic illustration of the sector-specific distance tracking. The *Left* panel shows a Bayesian network representation of a dynamic generative model (i.e., a time-series model), that represents the time-evolution of a latent variable $x_{1,...,T}$ and simultaneous observations $y_{1,...T}$. Shown are both a standard time-series representation (*Lower Left*) and its equivalent representation as generalized coordinates of motion ${\tilde{x}}_{t}=\left(x_{t},x_{t}^{\prime },x_{t}^{\prime \prime },...\right)$ (*Right*). We show the orders of differentiation used for our model in practice (3 orders of motion for $\tilde{x}$ and 2 orders of motion for $\tilde{y}$). The *Middle* panel of *B* shows how each component of the vectorial hidden state $x=(x_{h,1},...,x_{h,L})$ is computed as the average nearest-neighbor distance for the neighbors within each visual sector. Observations are generated as noisy, Gaussian samples centered on the sector-wise distance hidden state (*Right* panel of *B*). This requires the agent to estimate the true hidden state $x_{t}$ by performing inference with respect to a generative model of how sensory data are generated $p(\tilde{y},\tilde{x})$.

In contrast, the active inference approach forgoes specifying explicit vectorial forces, and instead starts by modeling all behavior as the solution to an inference problem, namely the problem of inferring the latent causes of sensations. Perception and action strive to improve the agent’s predictions of sensory inputs, based on its internal model of its world (Fig. 1*A*). By equipping this internal model with expectations about the environment’s underlying tendencies, social forces can emerge naturally as agents attempt to suppress sensory data that are mismatched with their expectations. This perspective shift offers a unifying modeling ontology for describing adaptive behavior, while also resonating with cybernetic principles like homeostatic regulation and process theories of neural function like predictive coding (26–29).

Active inference blends the construct validity of cognitivist approaches with the first-principles elegance of physics-based approaches by invoking minimization of a single, all-encompassing objective function that explains behavior: surprise, or, under certain assumptions, prediction error. As an example of this perspective shift, in this work, we investigate a specific class of generative models that can be used to account for the types of collective behaviors exhibited by animal groups. In doing so, we hope to showcase the benefits of the framework, while also proposing a testable model class for use in studies of biological collective motion.

## Active Inference and Generative Models of Behavior

A common pipeline in the quantitative study of animal behavior involves selecting a candidate behavioral algorithm or decision rule that may explain a given behavior and then fitting the parameters of the candidate model to experimental or observational data (16, 30). While these approaches often yield strong quantitative fits to data, the explanatory power of the models reduces to the interpretation of hard-coded parameters, which often have opaque relationships to real biological mechanisms or constructs (31).

In the active inference framework, we rather ask: What is the minimal model an organism might have of its environment that is sufficient to explain its behavior? Behavior is cast as the process by which the agent minimizes surprise or prediction error, with respect to this model of the world (22, 32). The principle of prediction-error minimization enjoys empirical support in neuroscience (26, 33) and a theoretical basis in the form of the Free Energy Principle (22, 23, 25), an account of all self-organizing systems that casts them as implicit models of their environments, ultimately in the service of minimizing the surprise (a.k.a., self-information) associated with sensory states (34–36).

What states-of-affairs count as surprising hinges on a generative model that can assign a likelihood to sensory data? When it comes to modeling behavior driven by this principle, the challenge then becomes specifying a generative or world model, whereby a particular pattern of behavior simply emerges by minimizing surprise.

According to active inference, agents minimize surprise by changing their beliefs about the world (changing which observations are considered surprising) or by acting on the world to avoid surprising sensory data. The former strategy is thought to correspond to passive processes such as perception and learning, whereas the latter corresponds to processes like active sensing and movement. Action is thus motivated by the desire to generate sensations that are as least surprising as possible.

In this paper, we describe the motion of mobile, mutually sensing agents as emerging from a process of collective active inference, whereby agents both estimate the hidden causes of their sensations, while also actively changing their position in space in order to minimize prediction error. In contrast to models that use prespecified behavioral rules for generating behavior, generative models entail collective behavior by appealing to a probabilistic representation of how an organism’s sensory inputs are generated.

## A Generative Model for a (Social) Particle

We now consider a sufficient generative model for an individual in a moving group. We equip this individual, hereafter referred to as the focal agent, with a representation of a simple random variable: the local distance x between itself and its neighbors. For generality, we can expand this into a multivariate random variable to describe a set of distances $x=(x_{1},x_{2},...,x_{L})$ that track the distance between the focal agent and its neighbors within L different sensory sectors (Fig. 1*B*). We analogize these L sectors to adjacent visual fields of an agent’s field of view (37, 38).

The focal agent possesses a model of the distance(s) x and its sensations thereof y. In particular, our focal agent represents the dynamics of x using a stochastic differential equation (a.k.a., a state-space model) defined by a drift f and some stochastic forcing ω—we refer to this component of the generative model as the dynamics model. The stochastic term ω captures the agent’s uncertainty about paths of x over time. The agent also believes it can sense x via observations y, mediated by a sensory map, which we call the observation model. This is defined by some (possibly nonlinear) function g with additive noise z. The agent’s generative model is then fully described by a pair of equations that detail 1) the time-evolution of the distance and 2) the simultaneous generation of sensory samples of the distance:

[1] $$\begin{array}{c} D\tilde{x}=\tilde{f}+\tilde{\omega }\;\;\tilde{y}=\tilde{g}+\tilde{z}. \end{array}$$

All random variables are described using generalized coordinates of motion with the convention $\tilde{q}=\left\{q,q^{\prime },q^{\prime \prime },...\right\}$. Generalized coordinates allow us to represent the trajectory of a random variable using a vector of local time derivatives (position, velocity, acceleration, etc.). The matrix D is a generalized derivative operator that moves a vector of generalized coordinates up one order of motion $D{\left(x,x^{\prime },x^{\prime \prime },...\right)}^{⊤}={\left(x^{\prime },x^{\prime \prime },x^{\prime \prime \prime },...\right)}^{⊤}$. The generalized functions $\tilde{f}$ and $\tilde{g}$ therefore operate on vectors of generalized coordinates (see *SI Appendix*, section S1 for details on generalized coordinates and filtering).

## Generalized Filtering and Active Inference

An agent equipped with this dynamic generative model then performs active inference by updating its beliefs (state estimation, or filtering) and control states (action) to minimize surprise.

Inference entails updating a probabilistic belief over hidden states $\tilde{x}$ in the face of sensory data $\tilde{y}$. Our agents solve this filtering problem using generalized filtering (39, 40), an algorithm for approximate Bayesian inference and parameter estimation on dynamic state-space models. This is achieved by minimizing the variational free energy F, a tractable upper bound on surprise (i.e., negative log evidence or marginal likelihood). The agent minimizes the free energy with respect to a belief distribution $q(\tilde{x})$ with parameters ν; this approximates the true posterior $q_{\nu }\left(\tilde{x}\right)\approx p\left(\tilde{x}|\tilde{y}\right)$, which is the optimal solution in the context of Bayesian inference. The true posterior $p(\tilde{x}|\tilde{y})$ is difficult to compute for many generative models due to the difficult calculation of the marginal (log) likelihood $\ln p(\tilde{y})$. Variational methods circumvent this intractable marginalization problem by replacing it with a tractable optimization problem: namely, adjusting an approximate posterior to match the true posterior by minimizing F with respect to its (variational) parameters ν.

We parameterize $q(\tilde{x})$ as a Gaussian with mean-vector $\tilde{\mu }$, which is a natural choice for this generative model since the assumption of normally distributed noises $\tilde{z},\tilde{\omega }$ imply that the true posterior will be Gaussian near the posterior mode $\arg\max p(\tilde{x}|\tilde{y})$. The implicit Gaussian (i.e., Laplace) assumption is ubiquitous in the modeling and signal processing literature (41) and can be regarded as a “minimal” assumption, by appeal to things like the central limit theorem and related principles (e.g., Jaynes’ maximum entropy principle). According to generalized filtering, $\tilde{\mu }$ is updated using a sum of prediction errors:

[2] $$\begin{array}{c} \frac{d\tilde{\mu }}{dt}\propto -\nabla_{\tilde{\mu }}F\left(\tilde{\mu },\tilde{y}\right)\\ \propto {\tilde{\varepsilon }}_{z}-{\tilde{\varepsilon }}_{\omega },\\ where\;\;{\tilde{\varepsilon }}_{z}=\tilde{y}-\tilde{g}(\tilde{\mu })\\ {\tilde{\varepsilon }}_{\omega }=D\tilde{\mu }-\tilde{f}(\tilde{\mu }). \end{array}$$

The ensuing evidence accumulation can be regarded as a natural generalization of predictive coding (26, 42, 43), where beliefs about local trajectories $\tilde{\mu }$ are updated using a running assimilation of sensory and model prediction errors: ${\tilde{\varepsilon }}_{z}$ and ${\tilde{\varepsilon }}_{\omega }$, respectively. For notational clarity, we have omitted terms that weigh these prediction errors; the so-called generalized sensory and model precisions ${\tilde{\Pi }}^{z},{\tilde{\Pi }}^{\omega }$, which encode the agent’s assumptions about the magnitude and correlation structure of noise. The importance of these precisions will become clear later, when understanding the relationship between precision-weighted prediction errors and social forces.

While inference entails changing the approximate posterior means $\tilde{\mu }$ to best explain sensory data, action entails changing the data itself to better match the data to one’s current beliefs. Similar to the update scheme in Eq. **2**, actions are also updated by minimizing free energy:

[3] $$\begin{array}{c} \frac{da}{dt}=-\nabla_{a}F(\tilde{\mu },\tilde{y}(a))\\ =-\nabla_{\tilde{y}}F(\tilde{\mu },\tilde{y}(a))\nabla_{a}\tilde{y}(a)\\ \propto -{\tilde{\varepsilon }}_{z}^{⊤}\nabla_{a}\tilde{y}(a). \end{array}$$

Actions thus are updated using a product of sensory prediction errors ${\tilde{\varepsilon }}_{z}$ and a “sensorimotor contingency” $\nabla_{a}\tilde{y}\left(a\right)$ or reflex arc. This sort of “reflexive action”—where control is simply targeted at minimizing sensory prediction errors—underlies active inference accounts of motor control (27, 44), and can be formally related to proportional-integral-derivative (PID) control (45). These prediction errors measure how far an agent’s observations are from its expectations; the agent then acts using Eq. **3** to minimize this deviation. Active inference agents are thus driven to act in a way that aligns with their (biased) expectations about the world (46). In the next section, we will see how building a particular type of bias into each agent’s generative model leads to the appearance terms in Eq. **3** that resemble social forces.

## Social Forces as a Consequence of Predictive Control

In particular, we take the agent’s action to be its heading direction a=v and examine the case where the agent observes the distance to its neighbors within a single sensory sector, i.e., L=1, $x=(x_{1})$. We distinguish the agent’s representation of the distance x from the actual distance using the subscript h. Therefore, $x_{h}=\left(x_{h,1},x_{h,2},...,x_{h,L}\right)$ denotes the average distances (and corresponding sensory samples $y_{h}$) calculated using the actual positions of other agents. For the case of L=1, and assuming the agent observes both the distance and its rate of change $y_{h,1}^{\prime }$, this is,

[4] $$\begin{array}{cccc} x_{h,1} & =\frac{1}{K}\sum_{j\in N_{\mathrm{in}}}\left\|r_{j}-r\right\| & y_{h,1} & =x_{h,1}+z_{h,1},\\ x_{h,1}^{\prime } & =\frac{dx_{h,1}}{\mathrm{dt}} & y_{h,1}^{\prime } & =x_{h,1}^{\prime }+z_{h,1}^{\prime }. \end{array}$$

$N_{\mathrm{in}}$ is the set of neighbors within the agent’s single sensory sector, K is the size of this set, r is the focal agent’s position vector, and $r_{j}$ is the position vectors of neighbor j. The sensory observation of the generalized distance ${\tilde{y}}_{h}=\left(y_{h,1},y_{h,1}^{\prime }\right)$ is a sample of the hidden state, perturbed by some additive noises $\tilde{z}=\left(z_{h,1},z_{h,1}^{\prime }\right)$. By expanding the active inference control rule in Eq. **3**, we arrive at the following differential equation for the heading vector:

[5] $$\begin{array}{cc} \frac{dv}{\mathrm{dt}} & =\xi_{z}^{\prime }\Delta \hat{r},\\ \xi_{z}^{\prime } & =\pi_{z,1}^{\prime }\left(y_{h,1}^{\prime }-\mu_{h,1}^{\prime }\right),\\ \Delta \hat{r} & =\frac{1}{K}\sum_{j\in N_{\mathrm{in}}}\frac{\Delta r_{j}}{\|\Delta r_{j}\|},\Delta r_{j}=r_{j}-r. \end{array}$$

The average vector $\Delta \hat{r}$ is exactly the (negative) sensorimotor contingency term $\nabla_{a}\tilde{y}\left(a\right)$ from Eq. **3** (see *SI Appendix*, section S1 for detailed derivations):

[6] $$\begin{array}{cc} \nabla_{v}\tilde{y}\left(v\right) & =\nabla_{r}y=\frac{1}{K}\sum_{j\in N_{\mathrm{in}}}\frac{r-r_{j}}{\|r-r_{j}\|}=-\Delta \hat{r}. \end{array}$$

The simple action update in Eq. **5** means that the focal agent moves along a vector pointing toward the average position of its neighbors. Whether this movement is attractive or repulsive is determined by the sign of the precision-weighted prediction error $\xi_{z}^{\prime }=\pi_{z,1}^{\prime }\left(y_{h,1}^{\prime }-\mu_{h,1}^{\prime }\right)$, and its magnitude depends on two factors: 1) the sensory precision or “reliability” $\pi_{z,1}^{\prime }$ that the agent affords observations of the rate-of-change of $y_{h,1}$; and 2) the degree to which these rate-of-change observations deviate from their predicted value $y_{h,1}^{\prime }-\mu_{h,1}^{\prime }$.

The presence of both attractive and repulsive forces depends on the agent’s model of the distance dynamics, captured by the functional form of $\tilde{f}$. In particular, consider forms of $\tilde{f}$ that relax x to some attracting fixed point η>0. Equipped with such a stationary model of the local distance, inference dynamics (c.f., Eq. **2**) will constantly bias its predictions μ according to the prior belief that the distance is pulled to η. Given this biased dynamics model and the action update in Eq. **3**, such an agent will move to ensure that distance observations ${\tilde{y}}_{h}$ are equal to the fixed point η.

This action update shows immediate resemblance to the attractive and repulsive vectors common to social force-based models (4, 5, 7), which often share the following general form:

[7] $$\begin{array}{cc} F_{\mathrm{attr}} & \propto \sum_{j\in Z_{A}}\frac{r_{\mathrm{ij}}}{\|r_{\mathrm{ij}}\|},\\ F_{\mathrm{repul}} & \propto -\frac{1}{K}\sum_{j\in Z_{R}}\frac{r_{\mathrm{ij}}}{\|r_{\mathrm{ij}}\|}, \end{array}$$

where $Z_{A},Z_{R}$ refer to distance-defined zones of attraction or repulsion, respectively. In the active inference framework, these social forces emerge as the derivative of the observations with respect to action $\nabla_{a}\tilde{y}$, where the sign and magnitude of the precision-weighted sensory prediction error $\xi_{z}^{\prime }$ determines whether the vector is attractive (toward neighbors) or repulsive (away from neighbors). The transition point between attraction and repulsion is therefore given by η, the point at which prediction errors switch sign.

An important consequence of this formulation is that, unlike the action rule used in social force-based models, the “steady-state” solution occurs when all social forces disappear (when prediction errors vanish). In this case, the agent ceases to change its heading direction and adopts its previous velocity. This occurs when the agent’s sensations align with its (biased) predictions $y_{h,1}\approx \eta$. In classic SPP models, this is equivalent to the different social force vectors exactly canceling each other.

We can therefore interpret social force-based models as limiting cases of distance-inferring active inference agents, because one can conceive of social forces as just those forces induced by free energy gradients; namely, the forces that drive belief-updating. In the case of our active inference agents, attractive and repulsive forces emerge naturally when we assume a) agents model the local distance dynamics as an attractor with some positive-valued fixed point η; b) agents can act by changing their heading direction and c) agents observe at least the first time derivative of their observations (e.g., $y_{h,1}^{\prime }$, but see *SI Appendix*, section S1 for detailed derivations).

It is worth highlighting the absence of an explicit, vectorial alignment force in this model, consistent with experimental findings in two species of fish (12, 17). The heading vectors of neighbors are nevertheless implicitly incorporated into the calculation of first-order prediction errors $\xi_{z}^{\prime }$ via the first-order hidden state $x_{h,1}^{\prime }$ (c.f., Eq. **4** and *SI Appendix*, section S1 and Eq. **S40**). In particular, the $x_{h,1}^{\prime }$ (from which the observations $y_{h,1}^{\prime }$ are sampled) is equivalent to the “relative velocity” term used in so-called selective attraction and repulsion models, where the instantaneous rate at which neighbors approach or move away, is used to drive movement (47). However, explicit alignment forces as seen in the Vicsek model (6) and 2- and 3-Zone Couzin models (7, 48) can also be recovered if we assume agents have a generative model of the average angle between their heading vector and those of their neighbors (see *SI Appendix*, section S2 for derivations of alignment forces).

## Multivariate Sensorimotor Control

Having recovered social forces as free energy gradients in the case of a single sensory sector (L=1), we now revisit the general formulation of the generative model’s state-space, where the hidden variable x is treated as an L-dimensional vector state: $x=(x_{1},x_{2},...,x_{L})$, with correspondingly L-dimensional observations $y=(y_{1},y_{2},...,y_{L})$.

Specifically, we consider each $x_{l}$ to represent the average distance-to-neighbors within one of a subset of adjacent sensory sectors, where each sector is offset from the next by a fixed intersector angle (see Fig. 1*B* for a schematic of the multisector set-up). The rest of the generative model is identical; the agents estimate these distances (and their temporal derivatives $x_{l}^{\prime },x_{l}^{\prime \prime },...$) while changing their heading direction to minimize free energy. Following the same steps as in the case of a single sector, the resulting update rule for v is a weighted sum of “sector-vectors,” where generalized observations from each sector-specific modality ${\tilde{y}}_{l}$ are used to compute the prediction errors that scale the corresponding sector-vector. This generalizes the scalar-vector product in Eq. **5** to a matrix-vector product:

[8] $$\begin{array}{cc} \frac{dv}{\mathrm{dt}} & ={\tilde{\xi }}_{z}^{⊤}\Delta \hat{R},\\ \Delta \hat{R} & =-\left[\begin{array}{c} \nabla_{v}{\tilde{y}}_{1}\\ \nabla_{v}{\tilde{y}}_{2}\\ \vdots \\ \nabla_{v}{\tilde{y}}_{L} \end{array}\right], \end{array}$$

where now the (negative) sensorimotor contingency $-\nabla_{a}\tilde{y}=\Delta \hat{R}$ is a matrix whose rows contain the partial derivatives $\nabla_{v}{\tilde{y}}_{l}$ (i.e., the sector-vectors). Each sector vector is a vector pointing toward the average neighbor position within sector l.

## Numerical Results

Given a group of active inference agents—equipped with the generative models described in previous sections—it is straightforward to generate trajectories of collective motion by integrating each agent’s heading vector over time: ${\dot{r}}_{i}=v_{i},i\in \left\{1,2,...,N\right\}$ where N is the number of agents. We update all heading directions ${\left\{v_{i}\right\}}_{i=1}^{N}$ and beliefs ${\left\{{\tilde{\mu }}_{i}\right\}}_{i=1}^{N}$ in parallel via a joint gradient descent on their respective free energies:

[9] $$\begin{array}{cccc} {\dot{v}}_{1} & =-\nabla_{v_{1}}F\left({\tilde{\mu }}_{1},{\tilde{y}}_{1}\right) & {\dot{\tilde{\mu }}}_{1} & =-\nabla_{{\tilde{\mu }}_{1}}F\left({\tilde{\mu }}_{1},{\tilde{y}}_{1}\right)\\ {\dot{v}}_{2} & =-\nabla_{v_{2}}F\left({\tilde{\mu }}_{2},{\tilde{y}}_{2}\right) & {\dot{\tilde{\mu }}}_{2} & =-\nabla_{{\tilde{\mu }}_{2}}F\left({\tilde{\mu }}_{2},{\tilde{y}}_{2}\right)\\ & \vdots & \vdots \\ {\dot{v}}_{N} & =-\nabla_{v_{N}}F\left({\tilde{\mu }}_{N},{\tilde{y}}_{N}\right) & {\dot{\tilde{\mu }}}_{N} & =-\nabla_{{\tilde{\mu }}_{N}}F\left({\tilde{\mu }}_{N},{\tilde{y}}_{N}\right). \end{array}$$

For the simulation results shown here, each agent tracks the average distance $x_{l}$ within a total of L=4 sensory sectors that each subtend $60^{{}^{\circ}}$ (starting at $-120^{{}^{\circ}}$ and ending at $+120^{{}^{\circ}}$, relative to the focal agent’s heading direction) and observe the sector-specific distances calculated using all neighbors lying within 5.0 units of the focal agent’s position. Each agent represents the vector of local distances as a generalized state with 3 orders of motion: $\tilde{x}=\left\{x,x^{\prime },x^{\prime \prime }\right\}$, $\tilde{\mu }=\left\{\mu ,\mu^{\prime },\mu^{\prime \prime }\right\}$. Agents can observe the first and second orders of the distance $\tilde{y}=\left\{y,y^{\prime }\right\}$, i.e., the distance itself and its instantaneous rate-of-change. In the numerical results to follow, we use active inference to study the relationship between the properties of individual cognition (e.g., the parameters of agent-level generative models) and collective phenomenology.

### Collective Regimes.

**Fig. 2.**
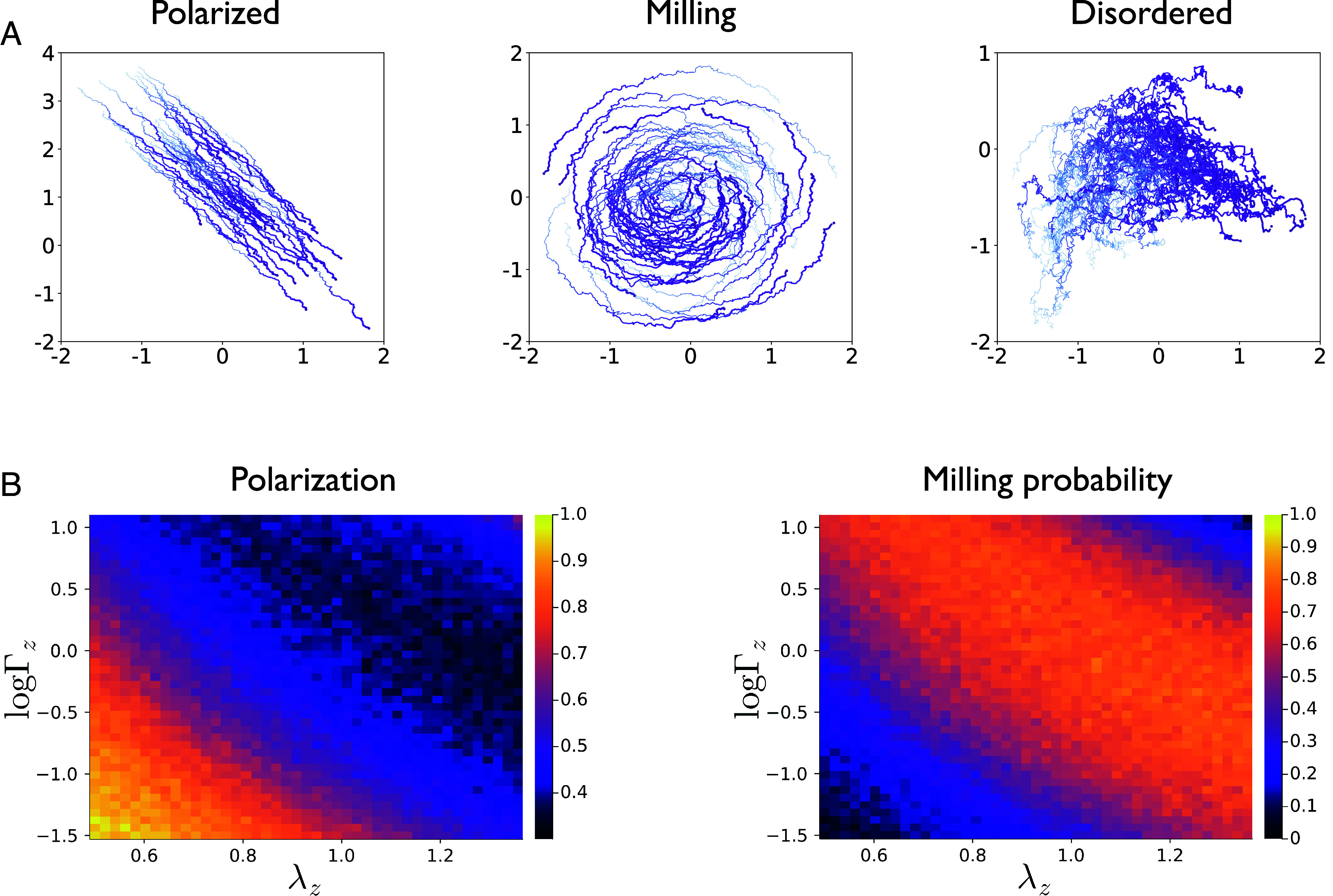
(*A*) Example snapshots of different collective states in schools of N=50 active inference agents. Each line represents the trajectory of one individual, and color gradient represents time, from earliest (light blue) to latest (purple). The polarized regime in the *Left* panel was simulated with the default parameters listed in *SI Appendix*, Table S1. The milling regime (*Middle* panel) was achieved by increasing the variance of velocity fluctuations (encoded in $\sigma_{z^{\prime },h}^{2}$) from 0.01 to 0.05 (relative to the default configuration) and increasing $\lambda_{z}$ from 1.0 to 1.2. The disordered regime was achieved by increasing the sensory smoothness parameter to 2.0 and decreasing η from 1.0 to 0.5 and α from 0.5 to 0.1 (relative to the default configuration). (*B*) Average polarization (*Left*) and milling probability (*Right*) shown as a function of the two factorized components of the sensory precision, $\Gamma_{z}$ (log-transformed) and $\lambda_{z}$. For each combination of precision parameters, we ran 500 independent trials of “free schooling,” and then averaged the quantities of interest across trials. Each free schooling trial lasted 15 s (1,500 time steps with dt=0.01s); the time-averaged metrics (polarization and milling probability, respectively, were computed from the last 10s of the trial.

Simulated groups of these distance-inferring agents display robust, cohesive collective motion (Fig. 2*A* and Movies S1–S5). Fig. 2*A* displays examples of different types of group phenomena exhibited in groups of active inference agents, whose diversity and types resemble those observed in animal groups (49, 50) and in other collective motion models (6, 7, 51). These range from directed, coherent movement with strong interagent velocity correlations (“polarized motion”) to group rotational patterns, like milling, which features high angular momentum around the group’s center of mass.

### Relating Individual Beliefs to Collective Outcomes.

In all but the most carefully constructed systems (31, 52, 53), the relationship between individual and collective representations is often opaque. In particular, the relationship between individual-level uncertainty or “risk” and collective behavior is an open area of research. For instance, some research has indicated that increased risk-sensitivity at the level of the individual may lead to decreased risk-encoding at the collective level (54). Inspired by these observations, we use active inference to examine the quantitative relationship between uncertainty at the individual-level and collective phenomenology. We begin by examining common metrics of group motion like polarization and angular momentum (7). In Fig. 2*B* we explore how polarization and angular momentum are affected by two components of agent-level sensory uncertainty (i.e., inverse sensory precision): 1) the absolute precision that agents associate with sensory noise and 2) the autocorrelation or “smoothness” associated to that noise.

These components are encoded in each agent’s observation model, which assumes generalized distance observations $\tilde{y}$ are normally distributed around the generalized state $\tilde{x}$:

[10] $$\begin{array}{c} P\left(\tilde{y}|\tilde{x}\right)=N\left(\tilde{y};\tilde{x},{\tilde{\Sigma }}^{z}\right), \end{array}$$

where we focus on the parameterization of the inverse of the covariance matrix, a.k.a., the precision matrix ${\tilde{\Pi }}^{z}={\left({\tilde{\Sigma }}^{z}\right)}^{-1}$. This precision matrix factorizes into two submatrices, one encoding the amplitude of random fluctuations z and one encoding their temporal smoothness, i.e., the inverse of the covariance between different derivatives of random fluctuations (e.g., between z and $z^{\prime }$):

[11] $$\begin{array}{c} {\tilde{\Pi }}^{z}=\Pi^{z}⊗{\tilde{\Pi }}^{z}\\ \text{where }\Pi^{z}=\left[\begin{array}{cccc} \Gamma_{z,1} & 0 & \cdots & 0\\ 0 & \Gamma_{z,2} & & \\ \vdots & \ddots & & \\ 0 & & \Gamma_{z,L} & \end{array}\right] \end{array}$$

[12] $${\tilde{\prod }}^{\text{z}}=\left[\begin{array}{cc} 1 & 0\\ 0 & 2\lambda_{z}^{2} \end{array}\right].$$

Intuitively, $\Gamma_{z}$ encodes the variance or amplitude that the agent associates with the noise in each of its L sensory sectors $z_{l}$, and $\lambda_{z}$ encodes how “smooth” the agent believes the noise is (40, 55). A higher value of $\lambda_{z}$ implies that the agent believes sensory noise is more serially correlated (e.g., random fluctuations in optical signals caused by smooth variations in refraction due to turbulence in water). *SI Appendix*, section S3 shows how the smoothness parameter $\lambda_{z}$ can be derived from a noise process with a Gaussian autocorrelation function. The consequences of this parameterization can be mapped back to the first-order prediction errors $\xi_{z}^{\prime }$ that drive action in Eqs. **5** and **8**:

[13] $$\begin{array}{cc} \xi_{z}^{\prime } & =\left[\begin{array}{c} 2\Gamma_{z,1}\lambda_{z}^{2}\left(y_{h,1}^{\prime }-\mu_{h,1}^{\prime }\right)\\ 2\Gamma_{z,2}\lambda_{z}^{2}\left(y_{h,2}^{\prime }-\mu_{h,2}^{\prime }\right)\\ \vdots \\ 2\Gamma_{z,L}\lambda_{z}^{2}\left(y_{h,L}^{\prime }-\mu_{h,L}^{\prime }\right) \end{array}\right]. \end{array}$$

Here, we have simply written the precision assigned to noise $z_{h,l}$ in a particular sensory sector as a product of the amplitude and smoothness parameters: $\pi_{z,l}^{\prime }=2\Gamma_{z,l}\lambda_{z}^{2}$.

Fig. 2*B* shows how the different components (amplitude and smoothness) of the agent’s beliefs about uncertainty determine group behavior, as quantified by average polarization and milling probability. Average polarization is defined here as the time average of the polarization of the group, where the polarization at a given time p(t) measures the alignment of velocities of agents comprising the group (7, 56):

[14] $$\begin{array}{c} \hat{p}=\frac{1}{T-t_{0}}\overset{T}{\underset{t=t_{0}}{\sum }}p(t)\;\;\;\;\;\;\;\;\;\;\;p(t)=\frac{1}{N}\|\overset{N}{\underset{i=1}{\sum }}v_{i}(t)\|. \end{array}$$

Note that the time average is calculated once steady state has been reached, where the beginning of this state is indicated by $t_{0}$ (for the heatmaps shown in Fig. 2*B*, we calculate these average metrics with $t_{0}=5$ s). High average polarization indicates directed, coherent group movement. The *Left* panel of Fig. 2*B* shows how $\Gamma_{z}$ and $\lambda_{z}$ contribute to the average polarization of the group. An increase in either parameter causes polarization to decrease and angular momentum to increase, reflecting the transition from directed motion to a milling regime, where the group rotates around its center of mass. We calculate the milling probability (c.f. *Right* panel of Fig. 2*B*) as the proportion of trials where the time-averaged angular momentum surpassed 0.5. The average angular momentum can be used to quantify the degree of rotational motion, and is calculated as the time- and group-average of the individual angular momenta around the groups’ center of mass c:

[15] $$\begin{array}{c} \hat{m}=\frac{1}{T-t_{0}}\overset{T}{\underset{t=t_{0}}{\sum }}m(t)\;\;\;\;\;\;\;\;\;m(t)=\frac{1}{N}\|\overset{N}{\underset{i=1}{\sum }}r_{ic}(t)\times v_{i}(t)\|, \end{array}$$

where $r_{\mathrm{ic}}$ is a relative position vector for agent i, defined as the vector pointing from the group center c to agent i’s position: $r_{i}-c$. We observed a large range of $\Gamma_{z}$ and $\lambda_{z}$ for which the milling regime (high average angular momentum) was stable (Fig. 2*B*, *Right*). This stands in contrast to earlier self-propelled particular models like the original 3-zone Couzin model, where milling was only stable under a relatively limited range of parameters (7).

These collective changes can be understood by recalling how first-order prediction errors $\xi_{z}^{\prime }$ (and thus the velocity update) depend on $\Gamma_{z}$ and $\lambda_{z}$:

[16] $$\begin{array}{cc} \xi_{z}^{\prime } & \propto 2\Gamma_{z}\lambda_{z}^{2}. \end{array}$$

In practice, this means that as the group believes in more predictable (less rough) first-order sensory information $y_{z}^{\prime }$, the group as a whole is more likely to enter rotational, milling-like regimes. However, the enhancing effect of these first-order prediction errors $\xi_{z}^{\prime }$ on rotational motion is bounded; if prediction errors are overweighted (e.g., high $\Gamma_{z}$ and/or $\lambda_{z}$), the group becomes more polarized again and likely to fragment (*SI Appendix*, Fig. S1). This fragmentation probability occurs at both low and high levels of $\Gamma_{z}$ and $\lambda_{z}$, implying that there is an optimal range of individual-level sensory precision where cohesive group behavior (whether polarized or milling) is stable. Thus, our model predicts that assuming one’s sensory information is highly precise is neither required, or in fact even desirable, for animals in order to facilitate collective motion.

We have seen how one can use active inference to relate features of individual-level beliefs (in this case, beliefs about sensory precision) to collective patterns, focusing in the present case on common metrics for studying collective motion like polarization and the tendency to mill.

In the following sections, we move from looking at group-level patterns that occur during free movement, to studying the consequences of individual-level uncertainty for collective information-processing. We begin by investigating how collective information transfer depends on individual-level beliefs about the relative precisions associated with different types of sensory information.

### Collective Information Transfer.

**Fig. 3.**
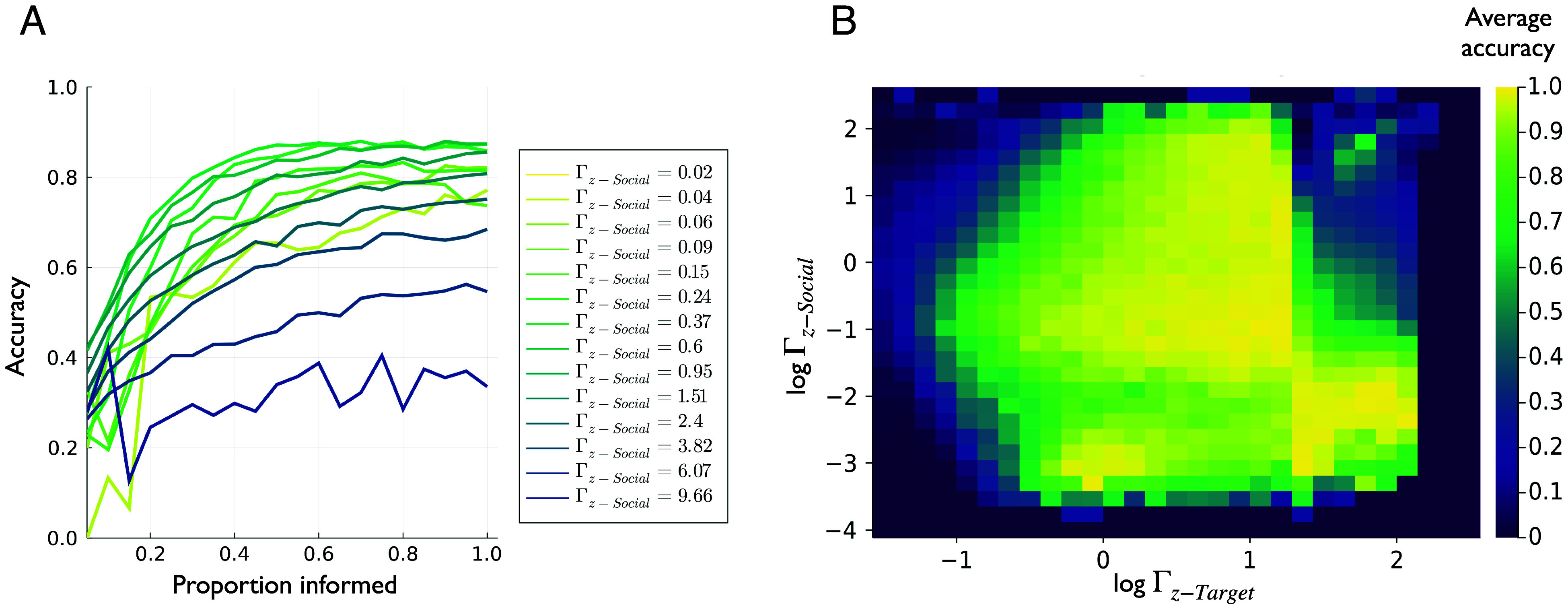
(*A*) Collective accuracy as a function of proportion informed or $p_{\inf}$ for differing values of the sensory precision assigned to social observations $\Gamma_{z-\text{Social}}$. Average accuracy for each condition (combination of $p_{\inf},\Gamma_{z-\text{Social}},\Gamma_{z-\text{Target}}$) was computed as the proportion of successful hits across 500 trials. Here, the average accuracy is further averaged across all the values of the $\Gamma_{z-\text{Target}}$ parameter, meaning each accuracy here is computed as the average of 15,000 total trials (500 trials per condition ×30 different values of $\Gamma_{z-\text{Target}}$). (*B*) Collective accuracy as a function of both the social and target precisions ($\Gamma_{z-Social},\Gamma_{z-Target}$, shown in log-scale) averaged across values of $p_{\inf}$ ranging from $p_{\inf}=0.15$ to $p_{\inf}=0.40$. Each condition’s accuracy was computed as the proportion of accurate decisions from 500 trials.

In this section, we take inspiration from the collective leadership and decision-making literature to investigate how individuals in animal groups can collectively navigate to a distant target (48, 57–59). This phenomenon is an example of effective leadership through collective information transfer and is remarkable for a number of reasons; one that speaks to its emergent nature, is the fact that these collective decisions are possible despite—and indeed even because of—the presence of uninformed individuals in the group (57). Fig. 3*A* shows that active inference agents engaged in this task reproduce a result from earlier work (48) on the relationship between the proportion of uninformed individuals and collective accuracy. Namely, as the proportion of informed individuals increases, so does the accuracy of reaching the majority-preferred target. In the same vein as earlier sections, we also investigated the dependence of this effect, as well as the average target-reaching accuracy, on individual-level beliefs.

We operationalize the notion of an agent being “informed” (about an external target) by introducing a new latent variable to its generative model; this variable $x_{\text{target}}$ represents the distance between the informed agent’s position r and a point-mass-like target with position vector $T=[T_{1},T_{2}]$. We thus define this hidden state and observation as follows: $x_{\text{target}}=\left\|T-r\right\|$, $y_{\text{target}}=x_{\text{target}}+z_{\text{target}}$. Just like the “social” distance observations $y_{h}$, this target distance observation $y_{\text{target}}$ represents a (potentially noisy) observation of the true distance $x_{\text{target}}$. As before, the agent represents both the target distance $x_{\text{target}}$ and its observations $y_{\text{target}}$ using generalized coordinates of motion. Each informed agent has a dynamics model of ${\tilde{x}}_{\text{target}}$, whereby they assume the target distance is driven by some drift function $f_{\text{target}}\left(x_{\text{target}}\right)=-\alpha_{t}x_{\text{target}}$ which relaxes to 0. As with the social distances, we truncate the agent’s generalized coordinates embedding of the target distance to three orders of motion and the generalized observations to two orders of motion.

Each informed agent maintains a full posterior belief $\tilde{\mu }=\left({\tilde{\mu }}_{1},{\tilde{\mu }}_{2},...,{\tilde{\mu }}_{L},{\tilde{\mu }}_{\text{target}}\right)$ about the local distances ${\tilde{x}}_{1},{\tilde{x}}_{2},...,{\tilde{x}}_{L}$ as well as the target distance ${\tilde{x}}_{\text{target}}$.

Using identical reasoning to arrive at the action updates in Eqs. **5** and **8**, one can augment the matrix-vector product in Eq. **8** with an extra sensorimotor contingency and prediction error that represents target-relevant information:

[17] $$\begin{array}{cc} \frac{dv}{\mathrm{dt}} & ={\tilde{\xi }}_{z}^{⊤}\left[\begin{array}{c} \Delta \hat{R}\\ \Delta T \end{array}\right]\\ \Delta T & =-\nabla_{v}{\tilde{y}}_{\;\text{target}}=\frac{T-r}{\left\|T-r\right\|}. \end{array}$$

This matrix-vector product can then be seen as a weighted combination of social and target vectors, with the weights afforded to each equal to their respective precision-weighted prediction errors:

[18] $$\begin{array}{cc} \frac{dv}{\mathrm{dt}} & =\underset{\text{Social vector}}{\underbrace{\xi_{\text{social}}\Delta \hat{R}}}+\underset{\text{Target vector}}{\underbrace{\xi_{\text{target}}\Delta T}}. \end{array}$$

This expression is analogous to the velocity update in equation 3 of ref. 48, where a “preferred direction” vector is integrated into the agent’s action update with some predetermined weight. This weight is described as controlling the relative strengths of nonsocial vs. social information. For active inference agents, the weighting of target-relevant information emerges naturally as a precision-weighted prediction error (here represented as $\xi_{\text{target}}$), and the target vector itself is equivalent to a sensorimotor reflex arc, that represents the agent’s assumptions about how the local flow of the target distance $y_{\text{target}}^{\prime }$ changes as a function of the agent’s heading direction v. An important consequence of this construction, is that, unlike in previous models where this weight is “baked-in” as a fixed parameter, the weight assigned to the target vector is dynamic, and fluctuates according to how much the agent’s expectations about the target distance ${\tilde{\mu }}_{\text{target}}$ predict the sensed target distance $y_{\text{target}}$.

Using this construction, we can simulate a group of active inference agents, in which some proportion $p_{\inf}$ of agents represent this extra set of target-related variables as described above. To generate ${\tilde{y}}_{\text{target}}$ observations for these informed individuals, we placed a spatial target at a fixed distance away from the group’s center-of-mass and then allowed the informed individuals to observe the generalized target distance ${\tilde{y}}_{\text{target}}=\left(y_{\text{target}},y_{\text{target}}^{\prime }\right)$. We then integrated the collective dynamics over time and measured the accuracy with which the group was able to navigate to the target (see *Materials and Methods* for details). By performing hundreds of these trials for different values of $p_{\inf}$, we reproduced the results of ref. 48 in Fig. 3. We see that as the number of informed individuals increases, collective accuracy increases. However, this performance gain depends on the agents’ beliefs about sensory precision, which we now dissociate into two components: $\Gamma_{z\text{-Social}}$ ( the precision assigned to the social distance observations) and $\Gamma_{z\text{-Target}}$ (the precision assigned to target distance observations). By varying these two precisions independently, which respectively scale $\xi_{\text{social}}$ and $\xi_{\text{target}}$ in Eq. **18**, we can investigate the dependence of collective accuracy on the beliefs of individual agents about the uncertainty attributed to different sources of information.

Fig. 3*A* shows the average collective accuracy as a function of $p_{\inf}$, for different levels of the social distance precision $\Gamma_{z\text{Social}}$. The pattern that emerges is that the social precision, that optimizes collective decision-making, sits within a bounded range. The general effect of social precision is to essentially balance the amplification of target-relevant information throughout the school, with the need for the group to maintain cohesion. When social precision is too high, agents overattend to social information and are not sensitive to the information provided by informed individuals; when it is too low, the group is likely to fragment and will not accurately share target-relevant information; meaning only the informed individuals will successfully reach the target. Fig. 3*B* shows that a similar optimal precision-balance exists for $\Gamma_{z\text{Target}}$. Here, we show average collective accuracy (averaged across values of $p_{\inf}$ as a function of social- and target-precision. Maximizing collective accuracy appears to rely on agents balancing the sensory precision they assign to different sources of information; under the active inference model proposed here, this balancing act can be exactly formulated in terms of the variances (inverse precisions) afforded to different types of sensory cues.

### Online Plasticity through Parameter Learning.

The ability of groups to tune their response to changing environmental contexts, such as rapid perturbations or informational changes, is a key feature of natural collective behavior (15, 54). However, many SPP models lack a generic way to incorporate this behavioral sensitivity (48) and exhibit damped, “averaging”-like responses to external inputs (60). This results from classical models usually equipping individuals with fixed interaction rules and constant weights for integrating different information sources. While online weight-updating rules and evolutionary algorithms have been used to adaptively tune single-agent parameters in some cases (48, 59, 61), these approaches are often not theoretically principled and driven by specific use-cases [with notable exceptions (62–64)].

Active inference offers an account of tunable sensitivity, using the same principle used to derive action and belief-updating in previous sections: minimizing surprise. In practice, this sensitivity emerges when we allow agents to update their generative models per se in real-time. Updating generative model parameters over time is often referred to as “learning” in the active inference literature (65), since it invokes the notion of updating beliefs about parameters rather than states, where parameters and states distinguish themselves by fast and slow timescales of updating, respectively. We leverage this idea to allow agents to adapt their generative models and thus adapt their behavioral rules, referring to this process as plasticity, in line with the notion of short-term plasticity in neural circuits (66). To enable agents to update generative model parameters, we can simply augment the coupled gradient descent in Eq. **9** with an additional dynamical equation, this time by minimizing free energy with respect to model parameters, which we subsume into a set θ:

[19] $$\begin{array}{cc} \dot{\theta } & =-\nabla_{\theta }F\left(\tilde{\mu },\tilde{y},\theta \right). \end{array}$$

The generative model parameters θ represent the statistical contingencies or regularities agents believe govern their sensory world; this includes the various precisions associated with sensory and process noises ${\tilde{\Pi }}^{z},{\tilde{\Pi }}^{\omega }$ and the parameters of the dynamics and observation models, $\tilde{f},\tilde{g}$. Since the free energy is a smooth function of all the generative model parameters, in theory, learning can be done with respect to any parameter using the procedure entailed by Eq. **19**.

In practice, combining parameter learning with active inference usually implies a separation of timescales, whereby learning or plasticity occurs concurrently to state inference and action but at a slower update rate. In all the results shown here, agents update parameters an order of magnitude more slowly than they update beliefs or actions. To furnish an interpretable example of plasticity, in the simulations described here, we enabled agents to update their beliefs about the sensory smoothness parameter $\lambda_{z}$. We chose sensory smoothness due to its straightforward relationship to the magnitude of sensory prediction errors (c.f. the relation in Eq. **16** and *SI Appendix*, section S3). As agents tune $\lambda_{z}$ to minimize free energy, belief updating and action will at the same time become quadratically more or less responsive to sensory information.

**Fig. 4.**
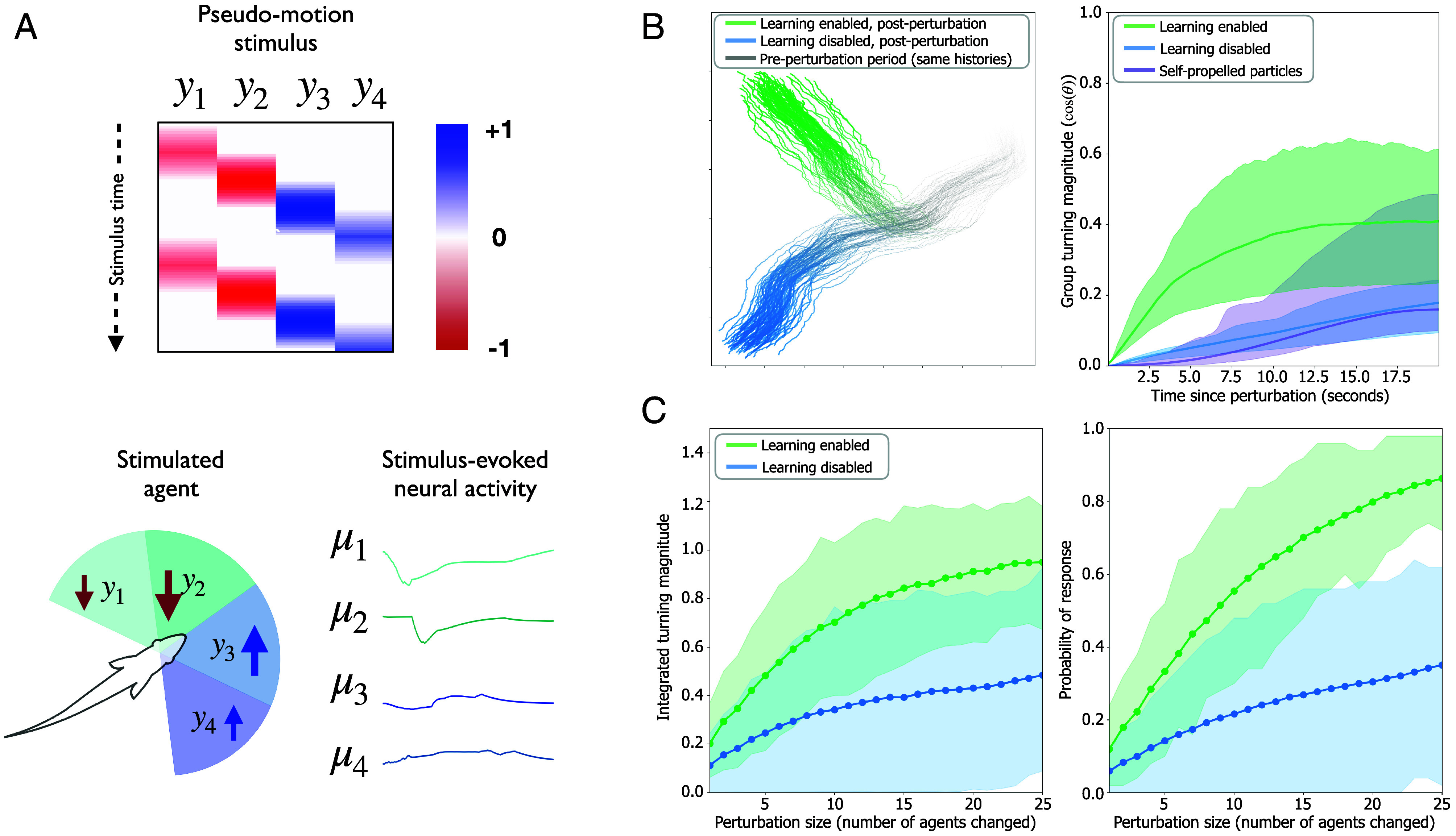
(*A*) Schematic of the sensory perturbation protocol. The “pseudomotion” stimulus consists of repetitively perturbing the agent’s sensory sectors with a moving wave of prediction errors in the agent’s velocity-observation modality $y_{h}^{\prime }$. The *Top* panel shows the stimulus pattern as a heatmap over (amplitude over time) with two repetitions, starting from negative (red, sectors 1 and 2) and transitioning to positive (blue, sectors 3 and 4) prediction errors. The sign-switch in the stimulus (from negative to positive) mimics a moving object that first moves toward focal individual and then moves away. The temporal order of the stimulus across the sectors can be used to selectively emulate a right-moving vs. left-moving object, relative to the focal individual’s heading-direction. The *Bottom* panel shows how the stimulated agent’s beliefs about the distance hidden state μ changes over the course of the motion stimulus, with these beliefs being analogized to hypothetical neural activity. (*B*) Response magnitude to a perturbation in the presence or absence of parameter learning. *Left* panel: example pair of 2-D trajectories of active inference agents with matched preperturbation histories, in response to an individual perturbation. The ability to perform parameter learning is left on in one stochastic realization (green) and turned off in the other (blue), following the perturbation. *Right* panel: initialization-averaged collective responses (group turning angle) to perturbation of active inference agents when learning is enabled or disabled. The perturbation response of a 2-zone SPP model (purple line) based on ref. 48 is also shown for reference. (*C*) Collective response as a function of the number of perturbed individuals, comparing simulations where parameter learning is enabled to those where it’s disabled. Shown is the mean response with highest density regions (HDRs) of integrated turning magnitude within 500 to 1,000 ms of the perturbation (*Left*) and response probability (*Right*) computed from $N_{i}=200$ independent initializations of each condition. For each initialization, the average metric is computed across $N_{r}=50$ independent realizations that were run forward from the same point in time, following a sensory prediction error perturbation (to a randomly chosen set of perturbed agents). Response probability is computed as the proportion of independent realizations, per initialization, where the group turning rate exceeded π radians within the first 10 s of the perturbation.

One example of where behavioral plasticity is crucial for collective information processing is a group’s ability to rapidly amplify behaviorally relevant information, e.g., detecting the presence of a predator (67–69). To study the effect of behavioral plasticity on collective responsiveness, we perturbed single agents in groups of active inference agents while enabling or disabling online plasticity. We perturbed groups by inducing transient “phantom” prediction errors in random subsets of agents and measuring the resulting turning response of the group (see *Materials and Methods* for details). These prediction errors were structured (Fig. 4*A*) to mimic a transient visual stimulus, e.g., a loom stimulus or approaching predator (70), which reliably induces a sustained turning response in the chosen individual (60). Fig. 4 shows the effect of enabling plasticity on the size and sensitivity of collective responses to these perturbations. Not only do plasticity-enabled groups respond more strongly to perturbations of single-agents, compared to their plasticity-disabled counterparts (Fig. 4*B*), but the magnitude of the collective response is also more sensitive to the size of the perturbation (Fig. 4*C*). As has been measured in biological collectives (71), the plasticity-enabled groups collectively encode the size of perturbations with a higher dynamic range than plasticity-disabled controls.

The active inference framework provides a flexible and theoretically principled approach to modeling adaptive, collective behavior with tunable sensitivity, that eschews ad hoc update rules or expensive evolutionary simulations. The plasticity mechanism proposed here is not limited to updating beliefs about sensory smoothness: it can be extended to update beliefs about any model parameter using the same principle. The ability to adapt generative model parameters in real-time represents a promising avenue for future research in active inference and collective behavior and may lead to more biologically plausible hypotheses about the mechanisms underlying adaptive responses in the natural world.

## Discussion

We have proposed active inference as a flexible, cognitively inspired model class that can be used in the theoretical study of collective motion, as well as in empirical settings as an individual-level model of behavior. By framing behavior as the consequence of prediction-error minimization—with respect to an individual’s world model—we offer examples of how naturalistic collective motion emerges in, where individual behavior is driven by the imperative to minimize the surprisal associated with sensory signals. Under mild distributional assumptions, this surprise is scored by an interpretable proxy; namely, prediction error. In the particular case of collective motion, a group of active inference agents equipped with a simple generative model of local social information can recover and generalize the social forces that have been the core mechanism in classical SPP models of collective motion. The active inference framework also provides a probabilistic interpretation of ad hoc “weight” parameters that are often used in these models, in terms of the precisions that agents associate with different types of sensory information.

We have also shown how the active inference framework can be used to characterize the relationship between generative model parameters and emergent information-processing capacities, as measured by collective information transfer and responsiveness to external perturbations. Active inference’s generality allows us to relax the typically static behavioral rules of SPP models, by enabling agents to flexibly tune their sensitivity to prediction errors. This is achieved via principled processes like parameter learning (i.e., “plasticity”), and can be used to model naturalistic features of collective behavior, such as the tendency to amplify salient (i.e., precise) information, that have largely evaded modeling in the SPP paradigm, except in cases where adaptation rules are explicitly introduced (48, 59). However, when we simply allow agents to update parameters, in addition to beliefs and agents, using the principle of surprise-minimization, many hallmarks of these naturalistic behaviors can be easily obtained.

The surprise minimization approach adopted here is both theoretically grounded in fundamental physical, cybernetic, and informational principles (23, 72–74) while also biologically inspired, due to the scalability of the belief and action update rules, which are hypothesized to be implementable on neuronal circuits (43). Our approach thus also harmonizes with modern “data-driven” approaches in behavioral biology, that aim to quantitatively estimate the behavioral algorithms used by different biological systems directly from experimental data (13–15).

By providing a flexible modeling approach that casts perception, action, and learning as manifestations of the single drive to minimize surprise, we have highlighted active inference as a toolbox for studying collective behavior in natural systems. Future work in this area could explore how the framework can be used to investigate other forms of collective behavior (not just collective motion), like multichoice decision-making, social foraging, and communication (75, 76). The results shown in the current work serve primarily as a proof of concept: we started by writing down a specific, hypothetical active inference model of agents engaged in group movement, and then generated naturalistic behaviors by integrating the resulting equations of motion (i.e., free energy gradients) for this particular model. Taking inspiration from fields like computational psychiatry (77, 78), we emphasize the ability to move from simple forward modeling of behavior to data-driven model inversion, whereby one hopes to infer the values of parameters that best explain empirical data (of e.g., behavioral movement data). Instead of using “force mapping” techniques to estimate social forces from behavioral measurements (79, 80), our approach would instead frame the problem as one of computational phenotyping, where alternative generative models that a particular animal might be equipped with, could be estimated from behavioral or neural data acquired from that animal. The resulting social forces or interaction rules would then emerge as those behaviors that minimize surprise, relative to the generative model that best explains the animal’s behavior. Both the estimation of model parameters and alternative model structures can be achieved through Bayesian model inversion and system identification methods like Bayesian model selection, averaging, or reduction (81).

## Materials and Methods

For all simulations we randomly initialized the positions and (unit-magnitude) velocities of N particles, and integrated the equations of motion for active inference and generalized filtering using a forward Euler–Maruyama scheme with an integration window of Δt=0.01s (see *SI Appendix*, section S6 for details). We varied group size N and the length of the simulation T (in seconds) depending on the experiment. Detailed background on generalized filtering, active inference, and derivations specific to the generative model we used for collective motion can be found in *SI Appendix*, section S1. All other parameters used for simulations, unless stated otherwise, are listed in *SI Appendix*, Table S1. The code (written in JAX and Julia) used to perform simulations can be found in the following open-source repository: https://github.com/conorheins/collective_motion_actinf.

### Quantifying Fragmented Groups.

For all experiments, we excluded trials where the group failed to maintain cohesion (or fragmented) to a sufficient degree. We deemed any given trial fragmented, when at least one individual was further than 2.0 dimensionless units away from all other individuals for at least 3 of the last 10 s of the trial. For the perturbation experiments, groups were excluded if this criterion was reached during the last 5 s of the 20 s postperturbation period.

### Collective Information Transfer Experiments.

For each trial of collective target-navigation, we initialized a group of N=30 agents with random positions and velocities (centered on the origin) and augmented the generative models of a fixed proportion $p_{\inf}$ of the total number of agents, where $p_{\inf}$ ranged from 0.05 to 1.0, with extra latent and observed variables representing the distance to the target with position vector T. The distance to the target was always 10 units from the origin. We measured collective accuracy as follows: we count a given trial as successful if the group is able to navigate to within 0.25 units of the target without losing cohesion within T=15 s (the length of each trial). The accuracy for a given experimental condition was then computed as the proportion of successes observed in 500 total trials.

### Perturbation Experiments.

For the perturbation experiments, we simulated $N_{i}=200$ randomly initialized independent runs of N=50 agents, which we term independent initializations. We ran each initialization forward for T=100 s, a point at which metrics like average polarization, angular momentum, and median nearest-neighbor distance were highly likely to have stopped changing and fluctuate around a stationary value. Starting at T=100 we then split each initialization into two further sets of $N_{r}=50$ parallel realizations. Each realization used a different random seed used to a) generate the action- and observation-noises; and b) select the candidate agent(s) for perturbation. Note that the splitting of seeds at T=100 means that each realization has an identical history up until that point. We enabled parameter learning of $\lambda_{z}$ in one set of realizations and we left it disabled in the other. We then perturbed random subsets of agents in both learning-enabled and -disabled realizations (2 to 50% of the group, i.e., 1 to 25 agents), by transiently inducing first-order prediction errors $\xi_{z}^{\prime }$ in the perturbed individuals (see *SI Appendix*, section S5 for perturbation details). We computed the relative group turning angle after the perturbation for 20 s to generate the plots in Fig. 4 *B* and *C*.

## Supplementary Material

Appendix 01 (PDF)

Movie S1. Example of a simulation of *N* = 96 agents that includes a dynamic transition from polarized to milling regime. Parameters: $\sigma_{z',h}^{2}=0.05$; Sector angle = 80°; *R*_0_ = 10 units; *κ_a_* = 0.2; λ*_ω_* = 0.5; λ*_z_* = 2.0. Unless specified, all remaining parameters are as listed in Table S1.

Movie S2. Example of a polarized group of *N* = 64 agents. Parameters: Sector angle = 80°;; *κ_a_* = 0.2; λ_*ω*_ = 0.5; λ*z* = 1.5.

Movie S3. Example of a milling regime observed in *N* = 64 agents. Parameters: $\sigma_{z',h}^{2}=0.04$; Sector angle = 80°; α = 1.0; *κ_a_* = 0.2; λ_*ω*_ = 0.8; λ*_z_* = 1.8.

Movie S4. Example of a disordered regime observed in *N* = 96 agents. Parameters: Number of sensory sectors = 2; Sector angle = 160°; *R*_0_ = 10.0 units; α = 0.2; η = 0.5, κ*_a_* = 0.2; λ_*ω*_ = 0.1; λ*_z_* = 1.787.

Movie S5. Metastable ‘snaking’ configuration observed in *N* = 64 agents. Parameters: $\sigma_{z',h}^{2}=0.04$; Sector angle = 80°; α = 0.1; *κ*_a_ = 0.2; λ_*ω*_ = 0.5; λ_z_ = 2.2.

## Data Availability

Github repository data have been deposited in https://github.com/conorheins/collective_motion_actinf (82).
