# Supplementary material for: Collective behavior from surprise minimization
Source: Proc Natl Acad Sci U S A. 2024 Apr 17;121(17):e2320239121. doi: 10.1073/pnas.2320239121 (PMC11046639; doi:10.1073/pnas.2320239121)
Supplement: Supplementary file 1 — Appendix 01 (PDF) [file pnas.2320239121.sapp.pdf]

PNAS

Supporting Information for

Collective behavior from surprise minimization

Conor Heins, Beren Millidge, Lancelot Da Costa, Richard P. Mann, Karl Friston, Iain D. Couzin

Conor Heins.
E-mail: cheins@ab.mpg.de

This PDF file includes:

Supporting text
Fig. S1
Table S1
Legends for Movies S1 to S5
SI References

Other supporting materials for this manuscript include the following:

Movies S1 to S5

Supporting Information Text

1. An active inference model of collective motion

Each agent within our model of collective motion maintains an internal model of its local environment represented by average distances to its neighbours. These distances are partitioned into L sensory sectors $\mathbf{x} = x_1, x_2, ..., x_L$, with each agent observing noisy versions of these distances through a corresponding sensory channel $\mathbf{y} = y_1, y_2, ..., y_L$. Each agent estimates the hidden distance variable(s) $\mathbf{x}$ over time using its observed sensory states $\mathbf{y}$. In practice, each agent implements this through a form of variational Bayesian inference developed for continuous data-assimilation in dynamic environments called *generalized filtering*, which can be seen as a variational, more flexible version of Kalman filters. This dynamic inference process entails updating posterior beliefs about $\mathbf{x}$ using a gradient descent on variational free energy. In the case of Gaussian assumptions about observation and state noise, these free energy gradients resemble a precision-weighted average of sensory and state prediction errors. This comprises the state-estimation component of active inference and is unpacked in detail in Section 1.

In addition to estimating the hidden distance variable with generalized filtering, each agent also changes its heading direction $\mathbf{v}$ in order to minimize the same variational free energy functional. When the agent's model of the distance dynamics is strongly 'biased' by a prior belief that the steady-state value of the distance variable(s) $\tilde{\mathbf{x}}$ hovers around a particular value $\boldsymbol{\eta}$, then agents will change their heading in a way that appears like they 'want' to maintain this target distance between them and their neighbours. Concretely, this means they move closer to neighbors when the sensed distance $\mathbf{y}$ is larger than expected, and move away from neighbors when $\mathbf{y}$ is smaller than expected.

This symmetry between belief updating and action, as both following the gradients of the same loss function, is what theoretically distinguishes active inference from other continuous control schemes, which often use different objectives for estimation and control. In the following sections we detail the processes of state-estimation and action under active inference.

Generalized filtering for dynamic models. Agents estimate hidden states x as the variational solution to a Bayesian inference problem; they achieve this in practice using an online-filtering algorithm known as generalized filtering (1, 2). Generalized filtering is a generic Bayesian filtering scheme for non-linear state-space models formulated in generalized coordinates of motion (3). It subsumes, as special cases, variational filtering (4), dynamic expectation maximization (5) and generalized predictive coding (6). This inversion scheme relies on a simple dynamical generative specification of hidden states x and how they relate to observations y. The generative model starts by postulating that the time evolution of a variable x is given by a stochastic differential equation with the following form:

$$\frac{dx_t}{dt} = f(x_t) + \omega_t \qquad [1]$$

where f is some deterministic flow function (i.e., a vector field) that depends on the current state x_t, and ω_t is a (smooth) additive Gaussian noise process. Under generalized filtering, we successively differentiate Eq. (1), to finesse the difficult computation of the *paths* or trajectories of x_t locally in time, by instead focusing on the much easier problem of computing the serial derivatives of x_t. This allows one to express a local trajectory of $\vec{x} = \{x_t, x_{t+1}, \ldots, x_{t+T}\}$ in terms of the derivatives of x_t, i.e., $\tilde{x}_t = (x'_t, x''_t, x'''_t, \ldots, x^{[n]}_t, \ldots)$, where $x^{[n]}_t := \frac{d^n}{dt^n} x_t$. We used the notation $\tilde{x}_t$ to denote a vector of these higher orders of motion at time t, a representation known as *generalized coordinates*. The equivalence between generalized coordinates and paths locally in time follows from Taylor's theorem, where the path of x around some time t can be expressed as a combination of its higher order derivatives:

$$x_{t+h} = x^{[0]}_t + \sum_{n=1}^{\infty} \frac{x^{[n]}}{n!} h^n \qquad [2]$$

Note that the (local in time) equality between a path $\vec{x}$ and its Taylor series only holds when the sample paths of x_t are analytic functions, which itself requires f to be analytic and the noise process ω_t to be analytic (in particular non-white noise fluctuations) (7). Successively differentiating the base equation in Eq. (1) (and ignoring contributions of the flow of order higher than one) yields a series of stochastic differential equations that describe the evolution of each order of motion $x^{[n]}_t$ as depending on its own state and the nth derivative of the noise (3):

$$\begin{aligned}
\dot{x} &= f(x) + \omega \\
\dot{x}' &= f_x x' + \omega' \\
\dot{x}'' &= f_x x'' + \omega'' \\
&\vdots \\
\Rightarrow D\tilde{x} &= \tilde{f} + \tilde{\omega}
\end{aligned}$$

where, following the notation used in (1–3), we use the notation f_x for the Jacobian (i.e., matrix of first order partial derivatives) of the flow function f evaluated at x, i.e., $Jf(x)$, and omit the time variable from our notation for conciseness.

Note that the above construction assumes a local linearization of f around x, in the sense that it ignores the contribution of higher order derivatives of the flow (3). When f is itself a linear function, this approximation is exact because contributions of the higher orders vanish. The D is the time derivative operator in generalised coordinates, with identity matrices along the first leading (block) diagonal and $\tilde{f}, \tilde{\omega}$ are the generalized flow function and generalized noises, respectively:

$$D = \begin{bmatrix} 0 & I & & \\ & \ddots & \ddots & \\ & & \ddots & I \\ & & & 0 \end{bmatrix} \qquad \tilde{f} = \begin{bmatrix} f(x^{[0]}) \\ f_x x^{[1]} \\ \vdots \\ f_x x^{[n]} \end{bmatrix} \qquad \tilde{\omega} = \begin{bmatrix} \omega^{[0]} \\ \omega^{[1]} \\ \vdots \\ \omega^{[n]} \end{bmatrix}$$

Here, n is some chosen order at which to truncate the derivatives. This truncation means that the Taylor expansion of a path $\vec{x}$ in Eq. (2) is rendered an approximation – valid locally in time. Having specified a dynamics over x (and its reformulation in generalized coordinates), we are in a position to specify the *observation model*. In generalized filtering, the generative model of state dynamics is supplemented with an observation model that maps hidden states x to their sensory consequences y via some (differentiable) sensory map $g(x)$ and additive Gaussian smooth fluctuations z:

$$y_t = g(x_t) + z_t \qquad [3]$$

Like the states, we can similarly express observations in generalized coordinates by successively differentiating Eq. (3) to obtain a similar single expression for the generalized observation equation:

$$\begin{aligned} y &= g(x) + z \\ y' &= g_x x' + z' \\ y'' &= g_x x'' + z'' \\ &\vdots \\ \Rightarrow \tilde{y} &= \tilde{g} + \tilde{z} \end{aligned}$$

where here the ith motion of observations $y^{[i]}$ is not a function of itself but rather that of the motion of the (generalized) hidden states $x^{[i]}$ and fluctuations $z^{[i]}$. In other words, the motion of observations tracks the simultaneous motion of the states, subject to any nonlinearities in the sensory map g and the motion of the noise z. Given Gaussian assumptions on the generalised noises $\tilde{\omega}$ and $\tilde{z}$, we can then write down the full hidden state and observation model $p(\tilde{y}, \tilde{x})$ in terms of Gaussian densities:

$$\begin{aligned} D\tilde{x} &= \tilde{f} + \tilde{\omega} \quad \tilde{\omega} \sim \mathcal{N}(\tilde{\omega}; \mathbf{0}, \tilde{\Sigma}^{\omega}) \\ \tilde{y} &= \tilde{g} + \tilde{z} \quad \tilde{z} \sim \mathcal{N}(\tilde{z}; \mathbf{0}, \tilde{\Sigma}^{z}) \\ \implies p(\tilde{y}, \tilde{x}) &= p(\tilde{y}|\tilde{x}) p(D\tilde{x}|\tilde{x}) \\ &= \mathcal{N}(\tilde{y}; \tilde{g}, \tilde{\Sigma}^{z}) \mathcal{N}(D\tilde{x}; \tilde{f}, \tilde{\Sigma}^{\omega}) \end{aligned} \qquad [4]$$

This Gaussian specification of the generative model licenses efficient, online update rules for the sufficient statistics of approximate posterior beliefs that track the expected value of the generalised hidden state $\tilde{x}$. This relies on a simple expression for the variational free energy of this state-space model; as we will see in the following sections, this not only enables efficient state estimation (a.k.a, updating beliefs about hidden states $\tilde{x}$), but also algorithms for inferring generative model parameters.

State estimation. Generalized filtering relies on optimizing posterior beliefs in order to minimize *variational free energy* F, an upper bound on the *surprise* associated with observations y under some generative model m:

$$F \geq \underbrace{-\ln p(y; m)}_{\text{surprise}} \qquad [5]$$

where the model m defines a joint distribution over observations and latent variables $p(y, \vartheta)$. The latent variables themselves ϑ are often split into hidden states x and parameters θ. Exact Bayesian inference entails obtaining the posterior distribution over latent variables $p(\vartheta|y)$, which can be expressed using Bayes rule:

$$p(\vartheta|y) = \frac{p(y, \vartheta)}{p(y)} \qquad [6]$$

$$p(y) \triangleq \int p(y, \vartheta) d\vartheta \qquad [7]$$

where hereafter we leave out the dependence on the model m.

In order to compute the posterior exactly, one has to compute the marginal probability of observations $p(y)$, also known as the marginal likelihood or model evidence. Computing the marginal likelihood is often intractable or difficult in practice, motivating the introduction of the variational bound, the free energy F, also known as the (negative) evidence lower-bound or ELBO. This can be shown by writing F as the Kullback-Leibler divergence between some "variational" distribution $q(\vartheta;\nu)$ over latent variables with parameters ν and the true posterior $p(\vartheta|y)$:

$$
\begin{aligned}
F &= \mathbb{E}_q\left[\ln q(\vartheta) - \ln p(y,\vartheta)\right] \\
&= D_{KL}\left(q(\vartheta;\nu)||p(\vartheta|y)\right) \underbrace{- \ln p(y)}_{\text{surprise}} \qquad [8]
\end{aligned}
$$

$$
\implies F \geq -\ln p(y) \qquad [9]
$$

The upper bound holds because the Kullback-Leibler divergence is always non-negative $D_{KL}(p||q) \geq 0$. Intuitively, as the variational distribution $q(\vartheta;\nu)$ better approximates the true posterior distribution $p(\vartheta|y)$, where the (in)accuracy of the approximation is measured by the KL divergence, then the tighter the free energy bounds the surprise. This decomposition also makes clear why minimizing F with respect to variational parameters ν is a way to update the variational distribution q to approximate the true posterior $p(\vartheta|y)$. The variational distribution is thus often referred to as an approximate posterior, where the exact posterior obtained by applying Bayesian rule as in Equation Eq. (6) corresponds to the variational posterior that minimises F.

Now we turn to deriving the Laplace-approximation to the variational free energy (VFE) for the state-space models used in generalised filtering. The Laplace approximation is an analytically tractable way to approximate the true posterior with a Gaussian distribution, which simplifies inference to an online filtering algorithm that corresponds to minimizing a sum of squared prediction errors.

Recall that our goal is to perform inference on the latent variables ϑ by optimizing an approximate posterior distribution $q(\vartheta;\nu)$. In our case, we let $\vartheta = \{x, \theta\}$ where x are hidden states and θ encompass other generative model parameters (e.g., hyperparameters of the generative model like $\tilde{f}, \tilde{g}, \tilde{\Sigma}^z, \tilde{\Sigma}^\omega$). For now we focus on inference over hidden states x and treat parameter inference later. Under the Laplace approximation we use a Gaussian distribution for the approximate posterior distribution $q(x;\nu)$:

$$
q(x;\nu) = \mathcal{N}(x; \underbrace{\mu, \Sigma^\nu}_{\nu}) \qquad [10]
$$

where the variational parameters ν are comprised of the sufficient statistics of a Gaussian distribution: the mean μ and covariance Σ^ν. We add the subscript ν to the variational variance to distinguish it from generative model covariances, e.g. $\tilde{\Sigma}^z, \tilde{\Sigma}^\omega$.

We can now arrive at a more specific expression for the variational free energy using the Gaussian form of the variational distribution. We start by decomposing the free energy into the sum of an expected energy term and a (negative) entropy, where the energy is defined as the negative log joint density over states and observations: $-\ln p(x,y)$ and the negative entropy is that of the variational posterior i.e., $\mathbb{E}_q[\ln q(x;\nu)]$:

$$
F = \mathbb{E}_q\left[-\ln p(x,y)\right] - \frac{1}{2}\left[\ln|\Sigma| + d\ln 2\pi e\right] \qquad [11]
$$

where d is the dimensionality of x and the full term on the right follows from the entropy of a multivariate Gaussian: $\mathrm{H}[\mathcal{N}(x;\mu,\Sigma)] = \frac{1}{2}\left[\ln|\Sigma| + d\ln 2\pi e\right]$.

Additional assumptions allow one to further simplify the expected energy term $\mathbb{E}_q\left[-\ln p(x,y)\right]$; namely, if we assume that the posterior is tightly peaked around the mean μ and that $p(x,y)$ is twice-differentiable in x, we can motivate a 2nd-order Taylor expansion of the expected energy term around its mode, i.e. when $x = \mu$:

$$
\begin{aligned}
\mathbb{E}_q\left[-\ln p(x,y)\right] &\approx \mathbb{E}_q\left[-\ln p(\mu,y) - \nabla_x \ln p(x,y)\Big|_{x=\mu}(x-\mu) - \frac{1}{2}(x-\mu)^\top \nabla_x^2 \ln p(x,y)\Big|_{x=\mu}(x-\mu)\right] \\
&= -\ln p(\mu,y) - \frac{1}{2}\operatorname{tr}\left(\Sigma\nabla_x^2 \ln p(x,y)\Big|_{x=\mu}\right) \qquad [12]
\end{aligned}
$$

Combining this approximation of the expected energy with the remaining terms in the variational free energy, we can now write the full expression of the Laplace-approximated free energy F_L:

$$
F_L = -\ln p(\mu,y) - \frac{1}{2}\operatorname{tr}\left(\Sigma\nabla_x^2 \ln p(x,y)\Big|_{x=\mu}\right) - \frac{1}{2}\left(\ln|\Sigma| + d\ln 2\pi e\right) \qquad [13]
$$

A useful feature of this expression is that the optimal variational covariance Σ^ν can obtained by setting the derivative of F_L with respect to the covariance Σ equal to 0 and solving for Σ, i.e. finding the values of the covariance that minimize the F_L:

$$\frac{\partial F_L}{\partial \Sigma} = 0 \iff \Sigma^\nu = -\left(\nabla_x^2 \ln p(x,y)\Big|_{x=\mu}\right)^{-1} \tag{14}$$

i.e., the optimal variance of the variational distribution is the curvature of the Laplace energy around its mode. Substituting this expression back into the full free energy, we can then write an expression that only depends on the mean vector μ of the variational density, since the variatonal variance Σ^ν is now expressed as a function of the mean:

$$\begin{aligned} F_L &= -\ln p(\mu, y) + \frac{1}{2}\underbrace{\mathrm{tr}\left(\Sigma^\nu (\Sigma^\nu)^{-1}\right)}_{=d} - \frac{1}{2}\left(\ln|\Sigma^\nu| + d\ln 2\pi e\right) \\ &= -\ln p(\mu, y) - \frac{1}{2}\left(\ln|\Sigma^\nu| + d\ln 2\pi\right) \end{aligned} \tag{15}$$

This means that the Laplace approximation to the variational free energy is a function of only the variational mean μ and sensory observations y, because the variational covariance Σ^ν is itself a function of μ. Belief updating then consists in minimizing the Laplace-approximated free energy F_L with respect to μ:

$$\dot{\mu} \propto -\nabla_\mu F_L(\mu, y) \tag{16}$$

Which consists of descending the gradient of the energy and log determinant terms with respect to μ. When the generative model $p(x, y)$ is Gaussian, the energy term is quadratic in μ and y. This means that its gradient can be written in terms of precision-weighted *prediction errors*, which score the difference between the expected observations (given the current value of μ) and the actual observations y. This notion of using prediction errors to estimate hidden quantities is also known as predictive coding (8–10). The log determinant term — in all of our cases of interest — turns out to have a vanishing gradient with respect to μ. In summary, the free energy gradient is a sum of precision-weighted *prediction errors*, and μ evolves to minimize those prediction errors.

To illustrate this, we take the simplest example — that of a linear, static, joint Gaussian generative model, where the prior over hidden states $p(x)$ is a Gaussian density with mean η and covariance Σ^ω, and the observation model $p(x|y)$ is a Gaussian density with mean $g(x)$, which is some linear function of the hidden state:

$$y \sim \mathcal{N}(g(x), \Sigma^z), \quad x \sim \mathcal{N}(\eta, \Sigma^\omega). \tag{17}$$

For this linear Gaussian generative model, the variational mean μ only influences the expected energy term of F_L, because the optimal covariance Σ^ν is independent of μ. Thus we ignore the constant entropy term and write out the energy as a sum of precision-weighted prediction errors:

$$\begin{aligned} -\ln p(\mu, y) &= -\ln p(y|\mu) - \ln p(\mu) \\ &= \frac{1}{2}\left[\varepsilon_z^T \Pi^z \varepsilon_z + \varepsilon_\omega^T \Pi^\omega \varepsilon_\omega\right] \\ &\text{where } \Pi^z = (\Sigma^z)^{-1}, \ \ \Pi^\omega = (\Sigma^\omega)^{-1} \\ &\text{and } \varepsilon_z = y - g(\mu), \ \ \varepsilon_\omega = \mu - \eta \end{aligned} \tag{18}$$

We can write out gradients of this quadratic energy function to yield the update equation for the means μ as in Eq. (16), and see that μ changes as a precision-weighted sum of 'sensory' and 'model' prediction errors (up to additive constants):

$$\begin{aligned} \dot{\mu} &= -\nabla_\mu F_L(\mu, y) \\ &= -\nabla_\mu \left[\frac{1}{2}\left(\varepsilon_z^T \Pi^z \varepsilon_z + \varepsilon_\omega^T \Pi^\omega \varepsilon_\omega\right)\right] \\ &= -\left[(g_\mu)^T \Pi^z \varepsilon_z + \Pi^\omega \varepsilon_\omega\right] \end{aligned} \tag{19}$$

Note that the variational means only depend on the terms of F_L containing ε_z and ε_ω, so that the update reduces to a gradient descent on a sum of squared prediction errors. This belief update scheme illustrates the key principles of predictive coding under the Laplace approximation: conditional means, denoted as μ, change as a function of precision-weighted prediction errors. The concept of precision-weighting in belief updating is intuitive: if the generative model attributes higher variance to sensory fluctuations as compared to state variance (i.e., $\Pi^z < \Pi^\omega$), then sensory data is relatively unreliable and consequently

makes a smaller impact on posterior beliefs. Therefore, the adjustment to the posterior mean μ in Eq. (19) is primarily influenced by the state prediction error term $\Pi^{\omega}\varepsilon_{\omega}$ or the prior. Conversely, when sensory information is allocated higher precision (lower variance) relative to prior beliefs (i.e., $\Pi^{z} > \Pi^{\omega}$), belief updates will strongly rely on sensory data.

We apply the above steps to derive the Laplace-approximated free energy with a Gaussian posterior $q(x;\nu)$ to the dynamical generative model in Eq. (4), which is constructed from Gaussian densities. Note that we use the tilde notation to now indicate that all variables are vectors of generalised coordinates, e.g., $\tilde{y}, \tilde{x}$, etc. Proceeding exactly as above, the Laplace free energy is a sum of the energy, a log determinant term, and a constant term, i.e. Eq. (15). Unlike in the linear Gaussian case, the potential non-linearity in the flows make that the log determinant term varies with respect to μ. However, as it turns out its gradient is approximately zero under the local linear approximation. Therefore, the only term that matters in the free energy as its gradient does not vanish is the energy term. In summary, we write:

$$
\begin{aligned}
F_L &\propto \tilde{\varepsilon}_z^T \tilde{\Pi}^z \tilde{\varepsilon}_z + \tilde{\varepsilon}_\omega^T \tilde{\Pi}^\omega \tilde{\varepsilon}_\omega \\
\tilde{\varepsilon}_z &\triangleq \tilde{y} - \tilde{g} \\
\tilde{\varepsilon}_\omega &\triangleq D\tilde{\mu} - \tilde{f}
\end{aligned}
\tag{20}
$$

Here, the so-called ‘generalised errors’ $\tilde{\varepsilon}_z$ and $\tilde{\varepsilon}_\omega$ encapsulate sensory and state prediction errors across orders of motion. Belief updating is again performed using a gradient descent on free energy, but the dynamic nature of inference necessitates an additional ’motion’ term:

$$
\begin{aligned}
\frac{d\tilde{\mu}}{dt} &= D\tilde{\mu} - \nabla_{\tilde{\mu}} F_L \\
&= D\tilde{\mu} + g_{\tilde{\mu}}^\top \tilde{\xi}_z + f_{\tilde{\mu}}^\top \tilde{\xi}_\omega - D^\top \tilde{\xi}_\omega \\
\text{where} \quad \tilde{\xi}_z &= \tilde{\Pi}^z \tilde{\varepsilon}_z \\
\tilde{\xi}_\omega &= \tilde{\Pi}^\omega \tilde{\varepsilon}_\omega
\end{aligned}
\tag{21}
$$

The additional term $D\tilde{\mu}$ places the gradient descent within the context of the expected movement of the conditional means $\tilde{\mu}$, and hence of the free energy minimum. This concept has been referred to as ’gradient descent in a moving frame of reference’ (1). This implies that free energy minimization does not occur when the beliefs cease moving, but rather when the belief update rate $\frac{d\tilde{\mu}}{dt}$ is identical to the beliefs about the motion itself $D\tilde{\mu}$, in other words when $\frac{\partial F}{\partial \tilde{\mu}} = 0 \iff D\tilde{\mu} = \frac{d\tilde{\mu}}{dt}$. This additional temporal correction proves beneficial in a dynamic data assimilation regime, where incoming observations are integrated online with beliefs that are evolving according to their own prior dynamics (1).

Active inference for continuous control. Active inference casts action or control as issuing from the same process of free energy minimization as used for state estimation; the only difference is that we now have an additional set of variables, actions a, that can be changed to minimize free energy as well. The update equation for actions a closely resembles that used to update the variational mean μ, i.e., a gradient descent on the (Laplace-encoded) variational free energy:

$$
\begin{aligned}
\frac{da}{dt} &= -\frac{\partial F_L(\mu, y(a))}{\partial a} \\
&= -\frac{\partial F_L}{\partial y(a)} \frac{\partial y(a)}{\partial a}
\end{aligned}
\tag{22}
$$

where we have now introduced a dependence between of observations y on actions a. This allows us to express the free energy gradient with respect to action as the product of the derivative of the free energy with respect to observations $\nabla_y F_L(\mu, y(a))$ and the derivative of the function mapping from actions to observations $\frac{\partial y(a)}{\partial a}$. The free energy gradient with respect to observations is exactly the sensory prediction error $\nabla_y F_L(\mu, y(a)) = \xi_z = \Pi(y - g(x))$. This assumed dependence of observations on actions underwrites the notion that active inference agents cannot directly measure how their actions affect hidden states, but may only do so via their sensory consequences. This has been speculated to explain the architecture of descending motor pathways in corticospinal systems, where motor commands are ‘unpacked’ into proprioceptive predictions at the level of spinal circuits and other lower motor nuclei. Action is thus realized by minimizing proprioceptive prediction errors via classical reflex arcs (11). The reflex arc term $\frac{\partial y(a)}{\partial a}$ of Eq. (22) is analogous to a forward model in motor control (12), because it reflects the agent’s implicit assumptions about how the agent’s own actions lead to their (anticipated) sensory consequences. This sort of update rule leads active inference agents to minimize sensory prediction errors via these ‘baked-in’ sensorimotor contingencies. In this way active inference has been referred to as ‘action by self-fulfilling prophecy’ (6). In other words, the agent generates top-down expectations of ‘preferred’ sensory inputs, which then generates prediction errors which can then be suppressed through low-level motoric reflexes (11).

Filtering and control for a self-propelled particle. Having derived a routine for state estimation and action through a generalized gradient flow on the Laplace-approximated variational free energy F_L, we can now apply this to the simulation of collective motion. In what follows, we write down a sufficient generative model for a single self-propelled agent and unpack the corresponding free energy gradients (Eq. (20) and Eq. (22)) using the structure and parameters of the chosen generative model. In this section we unpack the per-agent generative model of local distances described in the main text and demonstrate how a more parametric, unconstrained version of social forces are reproduced by minimizing free energy with respect to the distance-tracking generative model.

A generalised filter for local distances and their time evolution. As described in the main text, each agent represents a an L-dimensional vector $\mathbf{x}$ where $\mathbf{x} = (x_1, x_2, ..., x_L)$.[*] The agent not only represents the instantaneous value (or 'position') of $\mathbf{x}$ but also its generalized motion, which we truncate at 3rd order:

$$\begin{aligned}
\dot{\mathbf{x}} &= f(\mathbf{x}) + \boldsymbol{\omega} \\
\dot{\mathbf{x}}' &= f_{\mathbf{x}}\mathbf{x}' + \boldsymbol{\omega}' \\
\dot{\mathbf{x}}'' &= f_{\mathbf{x}}\mathbf{x}'' + \boldsymbol{\omega}'' \\
\Rightarrow D\tilde{\mathbf{x}} &= \tilde{\mathbf{f}} + \tilde{\boldsymbol{\omega}}
\end{aligned}$$

The flow at the first order f is a linear dynamical system with drift matrix A and fixed point with value $\boldsymbol{\eta}$:

$$f(\mathbf{x}) = -\mathbf{A}(\mathbf{x} - \boldsymbol{\eta}) \quad [23]$$

The eigenvalues of the $L \times L$ matrix $\mathbf{A}$ determine the rate at which the hidden states $\mathbf{x}$ are assumed to relax to their expected value of $\boldsymbol{\eta}$. In general, this matrix can be parameterized arbitrarily to encode different kinds of linear couplings among the different hidden states $x_1, x_2, ..., x_L$. In the present work we parameterize $\mathbf{A}$ simply as a diagonal matrix with a single diagonal value $\alpha > 0$, which can also be expressed as an α-scaled version of the identity matrix $L \times L$ identity matrix I_L:

$$\mathbf{A} = -\alpha I_L \quad [24]$$

In combination with the amplitude of random fluctuations Σ^{ω}, α determines how quickly the hidden states relax to their mean value of $\boldsymbol{\eta}$.[†] The generalised flow function $\tilde{\mathbf{f}}$ can thus be written as a linear function of the generalised state $\tilde{\mathbf{x}}$:

$$\begin{aligned}
\tilde{\mathbf{f}} = \begin{bmatrix} f(\mathbf{x}) \\ f_{\mathbf{x}}\mathbf{x}' \\ f_{\mathbf{x}}\mathbf{x}'' \end{bmatrix} &= -\begin{bmatrix} \mathbf{A} & \mathbf{0} & \mathbf{0} \\ \mathbf{0} & \mathbf{A} & \mathbf{0} \\ \mathbf{0} & \mathbf{0} & \mathbf{A} \end{bmatrix} \begin{bmatrix} \mathbf{x} - \boldsymbol{\eta} \\ \mathbf{x}' \\ \mathbf{x}'' \end{bmatrix} \\
&= \begin{bmatrix} -\alpha I_L & \mathbf{0} & \mathbf{0} \\ \mathbf{0} & -\alpha I_L & \mathbf{0} \\ \mathbf{0} & \mathbf{0} & -\alpha I_L \end{bmatrix} \begin{bmatrix} \mathbf{x} - \boldsymbol{\eta} \\ \mathbf{x}' \\ \mathbf{x}'' \end{bmatrix} = -\alpha \begin{bmatrix} \mathbf{x} - \boldsymbol{\eta} \\ \mathbf{x}' \\ \mathbf{x}'' \end{bmatrix} \quad [25]
\end{aligned}$$

where $\mathbf{0}$ are $L \times L$ matrices of zeros. We assume a multivariate Gaussian form for the generalized noises $\tilde{\boldsymbol{\omega}}$, meaning the density over the generalized motion $D\tilde{\mathbf{x}}$ is a Gaussian density, which we hereafter refer to as the 'dynamics model' or 'dynamical prior':

$$P(D\tilde{\mathbf{x}}|\tilde{\mathbf{x}}) = \mathcal{N}(D\tilde{\mathbf{x}}; \tilde{\mathbf{f}}, \tilde{\Sigma}^{\boldsymbol{\omega}}) \quad [26]$$

Consistent with the block diagonal form of the generalised flow function $\tilde{\mathbf{f}}$, we also assume the covariance of the generalized noises $\tilde{\Sigma}^{\boldsymbol{\omega}}$ factorizes into a Kronecker product of 'spatial' and 'temporal' covariance matrices, i.e.,

$$\tilde{\Sigma}^{\boldsymbol{\omega}} = \Sigma^{\boldsymbol{\omega}} \otimes \tilde{\Sigma}^{\omega} \quad [27]$$

where the spatial covariance $\Sigma^{\boldsymbol{\omega}}$ (note the bold superscript $\boldsymbol{\omega}$) represents covariance between L noise processes at the zero-th order $\boldsymbol{\omega}^{[0]}$, i.e., $\Sigma^{\boldsymbol{\omega}} = \mathbb{E}[\boldsymbol{\omega}^{[0]} \otimes \boldsymbol{\omega}^{[0]}]$, and $\tilde{\Sigma}^{\omega}$ encodes covariance between different derivatives of the first order noise, i.e., $\forall m, n : \left(\tilde{\Sigma}^{\omega}\right)_{nm} = \mathbb{E}[\omega^{[n]} \cdot \omega^{[m]}]$. The entries of this covariance matrix can be written in terms of the derivatives of the autocorrelation function of the random fluctuations evaluated at lag 0, $\rho(0)$:

[*] We use the bold notation $\mathbf{x}$ to represent a vector-valued variable

[†] Heuristically, it is an exponential decay rate.

$$\rho(h) \triangleq (\Sigma^{\boldsymbol{\omega}})^{-1}\mathbb{E}[\omega^{[0]}(\tau)\cdot\omega^{[0]}(\tau+h)]$$

$$\Rightarrow \tilde{\Sigma}^{\omega} = \begin{bmatrix} 1 & 0 & \ddot{\rho}(0) & \\ 0 & -\ddot{\rho}(0) & 0 & \\ \ddot{\rho}(0) & 0 & \ddddot{\rho}(0) & \\ & & & \ddots \end{bmatrix} \tag{28}$$

The checkerboard structure in the matrix reflects the fact that fluctuations at the first order are orthogonal to their motion (first derivative), but anti-correlated with their 2nd, 4th, ..., etc. derivatives. A derivation of the temporal covariance matrix from the autocorrelation function of the first-order fluctuations can be found in Appendix A.5.3 of (13). In the generative models of our agents, we assume a Gaussian autocorrelation function with "smoothness" parameter λ_ω, which yields a simple parameterization of $\tilde{\Sigma}^{\omega}$:

$$\rho(h) = e^{-\frac{h}{2\lambda_\omega}^2} \tag{29}$$

$$\Rightarrow \tilde{\Sigma}^{\omega} = \begin{bmatrix} 1 & 0 & -\frac{1}{2\lambda_\omega^2} & \cdots \\ 0 & \frac{1}{2\lambda_\omega^2} & 0 & \\ -\frac{1}{2\lambda_\omega^2} & 0 & \frac{3}{4\lambda_\omega^4} & \\ \vdots & & & \ddots \end{bmatrix} \tag{30}$$

A higher value of λ_ω dampens the variance of the generalised fluctuations at higher orders of differentiation. The correspondence of increasing λ_ω to an increasingly-autocorrelated process at the first order becomes intuitive once we consider the case of standard white noise, i.e., the derivative of the Wiener process, whose higher orders of motion have infinite variance (the state of the process at a given time changes infinitely quickly). This ability to handle differentiable noise goes beyond the usual Markovian assumptions made in standard state space models (e.g., Kalman-Bucy filters), which assume that the driving noise is white.

We parameterize the $L \times L$ spatial covariance $\Sigma^{\boldsymbol{\omega}}$ through its precision matrix $\Pi^{\boldsymbol{\omega}}$, as a diagonal matrix whose entries are given by a single precision (inverse variance) Γ_ω:

$$\Sigma^{\boldsymbol{\omega}} = (\Pi^{\boldsymbol{\omega}})^{-1} = \begin{bmatrix} \Gamma_\omega & 0 & 0 & \cdots \\ 0 & \Gamma_\omega & 0 & \\ 0 & 0 & \Gamma_\omega & \\ \vdots & & & \ddots \end{bmatrix}^{-1} \tag{31}$$

The observation likelihood describes sensory observations $\mathbf{y} = \{y_1, y_2, ..., y_L\}$ as noise-perturbed copies of the hidden states $\mathbf{x}$. We truncate generalized observations at second order, i.e., agents can sense the first order hidden state $\mathbf{x}$ and its motion $\mathbf{x}'$:

$$\begin{aligned} \mathbf{y} &= \mathbf{x} + \mathbf{z} \\ \mathbf{y}' &= \mathbf{x}' + \mathbf{z}' \end{aligned} \tag{32}$$

This can be equivalently expressed as a linear function $\tilde{\mathbf{g}}$ of the full generalised state $\tilde{\mathbf{x}} = \{\mathbf{x}, \mathbf{x}', \mathbf{x}''\}$, where $\tilde{\mathbf{g}}$ represents multiplication with a non-invertible matrix that discards acceleration information $\mathbf{x}''$:

$$\tilde{\mathbf{y}} = \tilde{\mathbf{g}} + \tilde{\mathbf{z}}$$

$$\begin{bmatrix} \mathbf{y} \\ \mathbf{y}' \end{bmatrix} = \begin{bmatrix} I_L & \mathbf{0} & \mathbf{0} \\ \mathbf{0} & I_L & \mathbf{0} \end{bmatrix} \begin{bmatrix} \mathbf{x} \\ \mathbf{x}' \\ \mathbf{x}'' \end{bmatrix} + \begin{bmatrix} \mathbf{z} \\ \mathbf{z}' \end{bmatrix} \tag{33}$$

We leverage the same assumptions about the sensory noises $\tilde{\mathbf{z}}$ as we did for the state noises $\tilde{\omega}$ to end up with the following multivariate Gaussian form for the observation model:

$$p(\tilde{\mathbf{y}}|\tilde{\mathbf{x}}) = \mathcal{N}(\tilde{\mathbf{y}}; \tilde{\mathbf{g}}, \tilde{\Sigma}^{\mathbf{z}}) \tag{34}$$

We parameterize the likelihood model's sensory noises $\tilde{\mathbf{z}}$ identically to the state noises $\tilde{\boldsymbol{\omega}}$, namely using a spatial precision parameter Γ_z and temporal smoothness parameter λ_z.

Having specified the dynamics and observation models in terms of Gaussian distributions, we can write out the full generative model as a joint Gaussian density over (generalized) hidden states and observations. We can furthermore define an approximate

posterior over the hidden states $\tilde{\mathbf{x}}$ that has a multivariate Gaussian form $Q(\tilde{\mathbf{x}}) = \mathcal{N}(\tilde{\mathbf{x}}; \tilde{\boldsymbol{\mu}}; \Sigma^\nu)$, which can be summarized entirely in terms of its posterior mean vector $\tilde{\boldsymbol{\mu}}$, due to the fact that under the Laplace approximation the variational covariance depends directly on the mean. From here, we can define the Laplace-approximated variational free energy for this generative model as proportional to a sum of squared prediction errors:

$$
\begin{aligned}
p(\tilde{\mathbf{y}}, \tilde{\mathbf{x}}) &= p(\tilde{\mathbf{y}}|\tilde{\mathbf{x}})p(D\tilde{\mathbf{x}}|\tilde{\mathbf{x}}) \\
&= \mathcal{N}(\tilde{\mathbf{y}}; \tilde{\mathbf{g}}, \tilde{\Sigma}^{\mathbf{z}})\mathcal{N}(D\tilde{\mathbf{x}}; \tilde{f}, \tilde{\Sigma}^{\boldsymbol{\omega}}) \qquad [35] \\
F_L &= \frac{1}{2}\left[\tilde{\varepsilon}_z^\top \tilde{\Pi}^{\mathbf{z}} \tilde{\varepsilon}_z + \tilde{\varepsilon}_\omega^\top \tilde{\Pi}^{\boldsymbol{\omega}} \tilde{\varepsilon}_\omega - \ln\left(|\tilde{\Pi}^{\mathbf{z}}||\tilde{\Pi}^{\boldsymbol{\omega}}||\Pi^\nu|\right) + 3L\ln 2\pi\right] \\
\text{where } \Pi^\nu &\triangleq (\Sigma^\nu)^{-1}
\end{aligned}
$$

$$
\tilde{\varepsilon}_z = \tilde{\mathbf{y}} - \tilde{\mathbf{g}}(\tilde{\boldsymbol{\mu}}) = \begin{bmatrix} \mathbf{y} - \boldsymbol{\mu} \\ \mathbf{y}' - \boldsymbol{\mu}' \end{bmatrix}, \quad \tilde{\varepsilon}_\omega = D\tilde{\boldsymbol{\mu}} - \tilde{\mathbf{f}}(\tilde{\boldsymbol{\mu}}) = \begin{bmatrix} \boldsymbol{\mu}' + \alpha(\boldsymbol{\mu} - \boldsymbol{\eta}) \\ \boldsymbol{\mu}'' + \alpha\boldsymbol{\mu}' \\ \alpha\boldsymbol{\mu}'' \end{bmatrix} \qquad [36]
$$

where the sensory prediction errors $\tilde{\varepsilon}_z$ score the difference between the generalized observations $\mathbf{y}, \mathbf{y}'$ and their expected values $\boldsymbol{\mu}, \boldsymbol{\mu}'$, and the model or process prediction errors $\tilde{\varepsilon}_\omega$ score the difference between the motion of the generalized means $D\tilde{\boldsymbol{\mu}}$ and their expected motion $\tilde{\mathbf{f}}(\tilde{\boldsymbol{\mu}})$, which has been expanded above using the linear form of the flow function detailed in Eq. (25). Note that here, due to the Laplace approximation, the generative model's expectation functions $\tilde{\mathbf{g}}, \tilde{\mathbf{f}}$ are evaluated at the variational mean $\tilde{\boldsymbol{\mu}}$, rendering the variational beliefs a moving point-estimate of the hidden states $\tilde{\mathbf{x}}$.

Filtering consists of updating $\tilde{\boldsymbol{\mu}}$ as a generalized gradient flow on this energy functional F_L as in Eq. (21). To be explicit, below we expand these free energy gradients using the particular forms of $\tilde{\mathbf{g}}, \tilde{\mathbf{f}}$ used by our self-propelled particle agent:

$$
\begin{aligned}
\frac{d\tilde{\boldsymbol{\mu}}}{dt} &= D\tilde{\boldsymbol{\mu}} - \nabla_{\tilde{\mu}} F_L \\
&= D\tilde{\boldsymbol{\mu}} + \nabla_{\tilde{\mu}}\tilde{\mathbf{g}}^\top \tilde{\boldsymbol{\xi}}_z + \nabla_{\tilde{\mu}}\tilde{\mathbf{f}}^\top \tilde{\boldsymbol{\xi}}_\omega - D^\top \tilde{\boldsymbol{\xi}}_\omega \\
\text{where } \tilde{\boldsymbol{\xi}}_z &= \tilde{\Pi}^{\mathbf{z}} \begin{bmatrix} \mathbf{y} - \boldsymbol{\mu} \\ \mathbf{y}' - \boldsymbol{\mu}' \end{bmatrix} \\
\tilde{\boldsymbol{\xi}}_\omega &= \tilde{\Pi}^{\boldsymbol{\omega}} \begin{bmatrix} \boldsymbol{\mu}' + \alpha(\boldsymbol{\mu} - \boldsymbol{\eta}) \\ \boldsymbol{\mu}'' + \alpha\boldsymbol{\mu}' \\ \alpha\boldsymbol{\mu}'' \end{bmatrix} \\
\nabla_{\tilde{\mu}}\tilde{\mathbf{g}} &= \begin{bmatrix} I_L & \mathbf{0} & \mathbf{0} \\ \mathbf{0} & I_L & \mathbf{0} \end{bmatrix}, \quad \nabla_{\tilde{\mu}}\tilde{\mathbf{f}} = \begin{bmatrix} -\alpha I_L & \mathbf{0} & \mathbf{0} \\ \mathbf{0} & -\alpha I_L & \mathbf{0} \\ \mathbf{0} & \mathbf{0} & -\alpha I_L \end{bmatrix} \qquad [37]
\end{aligned}
$$

This sort of filtering scheme means that the agent's beliefs $\tilde{\boldsymbol{\mu}}$ will evolve as a moving average of incoming sensory data $\tilde{\mathbf{y}}$ subject to a dynamical bias or "drag", which is a consequence of the latent belief that hidden states $\mathbf{x}$ continuously relax towards a fixed point at $\boldsymbol{\eta}$. Specifically, the beliefs are constantly pulled closer to the data in order to minimize sensory prediction errors $\tilde{\boldsymbol{\xi}}_z$; however, this process itself incurs state prediction errors $\tilde{\boldsymbol{\xi}}_\omega$ that will pull the beliefs back towards the fixed point. This constant tug of war between sensory and process prediction errors can be shifted disproportionately in one direction by adjusting the relative precisions of the likelihood vs. dynamical models, respectively. If the process precision $\tilde{\Pi}^{\boldsymbol{\omega}}$ is high relative to the observation precision $\tilde{\Pi}^{\mathbf{z}}$, then the beliefs will tend to their expected fixed point of $\boldsymbol{\eta}$. A similar enhancement of prior bias can be achieved by increasing the drift rate α of the dynamics model, which increases the force driving $\boldsymbol{\mu}$ towards $\boldsymbol{\eta}$ — this was the approach taken in (14), for example.

Note that when numerically integrating the differential equation in Eq. (37) with a forwards Euler scheme, one uses a finite number of iterations to update the variational means $\tilde{\boldsymbol{\mu}}$, which we term $n_{\text{InferIter}}$, and a step-size κ_μ which scales the size of the increment to $\tilde{\boldsymbol{\mu}}$ (6). In all simulations shown here, we set $n_{\text{InferIter}} = 1, \kappa_\mu = 0.1$ (see Table S1 for details).

Closing the loop with observations and action. In order to interpret the random variables of the generative model as representing behaviorally-relevant features of an agent's world, we now turn to specfiying the *generative process*, i.e., the actual physics of the world that our self-propelled particle agents will inhabit. In this section we detail how the observations $\tilde{\mathbf{y}}$ for a single agent are generated from the positions and velocities of other active inference agents, and how actions can be generated through *active inference*, which in this contexts means changing continuous control variables using a gradient descent on the same free energy used to derive the belief update equations of the previous section.

We now shift our perspective to that of a single agent, hereafter referred to as the *focal individual* or *focal agent*, and specify how its sensory data $\tilde{\mathbf{y}}$ are generated. We start by describing univariate hidden states and corresponding observations, where the true hidden variable is an average nearest-neighbor distance x_h. We add the h subscript to distinguish these 'real' variables (hidden states, observations, noise terms) from their representations in the generative model (e.g., $\tilde{x}$, $\tilde{y}$).

We indicate the focal individual with index i; so the agent i-relative hidden state $x_{h,i}$ denotes the average nearest-neighbor distance from the perspective of agent i. This average distance $x_{h,i}$ is calculated from the K neighbors that form the interaction

set N_{in} of the ith focal individual. How to define the interaction set N_{in} is a choice to make in each simulation, but for the case of recapitulating classical, distance-dependent social forces models, we define N_{in} as those neighbors that are within a fixed distance R_0 of the focal individual's position:

$$
\begin{aligned}
x_{h,i} &\triangleq \frac{1}{K} \sum_{j \in N_{in}} \|\Delta \mathbf{r}_{ij}\| \\
\text{where} \;\; N_{in} &\triangleq \{j \neq i \,:\, \|\Delta \mathbf{r}_{ij}\| \leq R_0\} \qquad [38] \\
K &\triangleq |N_{in}| \\
\Delta \mathbf{r}_{ij} &\triangleq \mathbf{r}_j - \mathbf{r}_i \qquad [39]
\end{aligned}
$$

An additional filter on N_{in} that is common to self-propelled particle models, is to only include neighbors that subtend some angular extent (also known as a 'vision cone' or 'visual field') relative to the focal agent's velocity vector $\mathbf{v}_i$. This is the approach taken in (15), for instance, and in the simulations examined in the main text we do the same.

The vector $\mathbf{r}_i$ denotes the 2-D coordinate of the focal agent, and $\mathbf{r}_j$ is that of neighbor j. $\mathbf{r}_{ij}$ thus represents the relative displacement vector of neighbour j, from the perspective of the focal agent i.

We also define the first temporal derivative of the local average distance $x'_{h,i}$:

$$
\begin{aligned}
\tilde{x}_{h,i} &\triangleq (x_{h,i}, x'_{h,i}) \\
x'_{h,i} &\triangleq \frac{dx_{h,i}}{dt} = \nabla_{\mathbf{r}_i} x_{h,i} \cdot \mathbf{v}_i + \sum_{j \in N_{in}} \left(\nabla_{\mathbf{r}_j} x_{h,i} \cdot \mathbf{v}_j\right) \qquad [40]
\end{aligned}
$$

where $\mathbf{v}_j$ is the velocity or heading vector of neighbour j. The expression in Eq. (40) means that we can compute the first derivative or velocity of the distance $x'_{h,i}$ as a function of the positions and velocities of all agents, as opposed to some discrete-time approximation, e.g., $x'_{h,i} \approx \frac{x_{h,i}(t+\Delta t) - x_{h,i}(t)}{\Delta t}$ for some small Δt. Note that this expression for $x'_{h,i}$ assumes a local linearization of $x_{h,i}$ at the radius defined by R_0, i.e., this linearization will be a poor predictor of the actual change in the state $x_{h,i}(t+\Delta t) - x_{h,i}(t)$ when neighbors are instantaneously leaving or entering the interaction set N_{in}. Observations $\tilde{y}_{h,i}$ are perturbed versions of the hidden states with additive generalised fluctuations $\tilde{z}_{h,i}$:

$$
\begin{aligned}
y_{h,i} &= x_{h,i} + z_{h,i} \\
y'_{h,i} &= x'_{h,i} + z'_{h,i} \\
\text{where} \quad p(\tilde{z}_{h,i}) &= N(\tilde{z}_{h,i}; \mathbf{0}, \tilde{\Sigma}_{z,h}) \qquad [41]
\end{aligned}
$$

In all simulations we parameterize the $\tilde{z}_{h,i}$ as independent Gaussian variables, i.e.,

$$
\tilde{\Sigma}_{z,h} = \begin{bmatrix} \sigma^2_{z,h} & 0 \\ 0 & \sigma^2_{z',h} \end{bmatrix} \qquad [42]
$$

where the two variances $\sigma^2_{z,h}$ and $\sigma^2_{z',h}$ can be set independently. The 'perception' step of our active inference process proceeds by providing these observations to the filtering equations in Eq. (37). The result is that posterior means $\tilde{\mu}$ appears to track $\tilde{x}_{h,i}$ over time, while additionally estimating its higher-order motion (acceleration) via μ''.

Finally, we now furnish a scheme for updating actions by mapping the control variables a and sensorimotor contingency terms of Eq. (22) to the case of our distance-tracking self-propelled agent.

We let actions be identifiable with the heading vector $\mathbf{v}_i$ of the focal individual, i.e., $a = \mathbf{v}_i$. For the simulations presented in the current paper, we always asserted that this heading have unit magnitude, but in general this constraint is not necessary.

Given this definition of actions, we can unpack the sensorimotor contingency term $\frac{\partial y(a)}{\partial a}$ that appeared in the active inference control equation of Eq. (22), now letting $a = \mathbf{v}$ and turning partial derivatives into Jacobians to account for vectorial nature of actions (being a velocity in 2-D) and observations (being comprised of two generalized coordinates):

$$
\frac{d\mathbf{v}_i}{dt} = -\nabla_{\mathbf{v}_i} \tilde{y}_{h,i}(\mathbf{v}_i)^\top \nabla_{\tilde{y}_{h,i}(\mathbf{v}_i)} F_L \qquad [43]
$$

Note here that observations $\tilde{y}_{h,i}$ are a function of actions; this is because observations are a linear function of hidden states, which themselves are linear in the velocity vector of the focal individual $\mathbf{v}_i$ via the relation in Eq. (40). Importantly, however, the distance observation $y_{h,i}$ does not directly depend on the $\mathbf{v}_i$ — only the distance velocity $y'_{h,i}$ does. This means the sensorimotor contingency in Eq. (43) is comprised of non-zero partial derivatives only for $y'_{h,i}$:

$$\nabla_{\mathbf{v}_i}\tilde{y}_{h,i}(\mathbf{v}_i) = \begin{bmatrix}\nabla_{\mathbf{v}_i} y_{h,i}(\mathbf{v}_i)\\ \nabla_{\mathbf{v}_i} y'_{h,i}(\mathbf{v}_i)\end{bmatrix} = \begin{bmatrix}\mathbf{0}\\ \nabla_{\mathbf{r}_i} x_{h,i}\end{bmatrix} \quad [44]$$

This has an important consequence for action, when we consider the form of the second part of the action update in Eq. (43), the free energy gradient term $\nabla_{\tilde{y}_{h,i}} F_L$:

$$\nabla_{\tilde{y}_{h,i}} F_L = \tilde{\xi}_z = \tilde{\Pi}_z \tilde{\varepsilon}_z = \begin{bmatrix}\Gamma_z(y_{h,i}-\mu)\\ 2\Gamma_z\lambda_z^2(y'_{h,i}-\mu')\end{bmatrix} \quad [45]$$

The free energy gradient with respect to observations is simply the generalized (precision-weighted) sensory error $\tilde{\xi}_z$, which we have written in terms of the observations $\tilde{y}_{h,i}$, posterior beliefs $\tilde{\mu}$ and precision parameters Γ_z, λ_z. The sparse form of the sensorimotor contingency in Eq. (44) means that the 0th-order prediction error ξ_z will have no effect on behavior and only the velocity prediction errors ξ'_z will be relevant for the update to $\mathbf{v}_i$, i.e.,

$$\begin{aligned}\frac{d\mathbf{v}_i}{dt} &= -\left(\xi_z \underbrace{\nabla_{\mathbf{v}_i} y_{h,i}(\mathbf{v}_i)}_{=\mathbf{0}} + \xi'_z \nabla_{\mathbf{v}_i} y'_{h,i}(\mathbf{v}_i)\right)\\ &= -\xi'_z \nabla_{\mathbf{r}_i} x_{h,i}\\ &= 2\Gamma_z\lambda_z^2(y'_{h,i}-\mu')\Delta\hat{\mathbf{r}}\\ \text{where} \quad \Delta\hat{\mathbf{r}} &= \frac{1}{K}\sum_{j\in N_{in}} \frac{\Delta\mathbf{r}_{ij}}{\|\Delta\mathbf{r}_{ij}\|} \end{aligned} \quad [46]$$

Note that, as for the inference update in Eq. (37), we update $\mathbf{v}_i$ using a fixed number of action iterations $n_{\text{ActionIter}}$ and step-size κ_a, where here we set $n_{\text{ActionIter}} = 1, \kappa_a = 0.1$. This action update equation has a few key implications for the behavior of active inference agents equipped with this type of generative model, and its relationship to 'classical' self-propelled particle models like the Couzin-Aoki model and the Reynolds or BOIDS model (15–17). The first is the fact that the sensorimotor contingency is identical to the 'social force' vector used to drive interactions in self-propelled particle models $\Delta\hat{\mathbf{r}}$; this the average of the vectors pointing from each neighbor in the interacting set to the focal agent's position $\mathbf{r}_i$. The sign of the precision-weighted prediction error ϵ'_z determines whether the social force is attractive (pointing towards other agents) or repulsive (pointing away from other agents). Secondly, the fact that actions only depend on velocity observations, rather than state observations, means that agents will adjust their heading according to how the (sensed) distance is instantaneously changing (its velocity), rather than its value. This lends action a predictive, anticipatory power and accounts for why we observe robust polarized motion in the absence of an explicit alignment term like in classic self-propelled particle models (15, 18). The alignment-like forces emerges from the fact that the velocity vectors of other agents $v_j, j \in N_{in}$ are integrated into the computation of $y'_{h,i}$ via the relation in the second line of Eq. (40).

One of the defining features of other self-propelled particle models like the Couzin-Aoki model (15, 16) is the presence and priorization of interaction zones. The two main zones used in these models, and which on their own are sufficient for group cohesion, are a narrow repulsion zone defined by some radius r_r and a wider attraction zone with radius r_a, where $r_a > r_r$. Neighboring agents within the repulsive radius exert repulsive forces on the focal agent, while those beyond the repulsion radius but within the attraction zone exert attractive forces, where the difference between attraction and repulsion is given by the sign of the force vector $\Delta\hat{\mathbf{r}}$. The active inference model leads to an effective notion of zones, but rather than being explicitly encoded, these zones emerge through the fixed-point attractor η parameterizing the generative model's dynamics model $\mathbf{f}$. This is made clear when we examine the precision-weighted prediction error ξ'_z, which itself is a function of velocity observations $y'_{h,i}$ and velocity beliefs μ'. Consider the limiting case of when inference is strongly biased by the dynamics model $\mathbf{f}$ (i.e., in the case that $\Gamma_\omega > \Gamma_z$ or large α); the generalised beliefs $\tilde{\mu}$ will be strongly drawn to the setpoint η of the dynamics prior, i.e.,

$$\tilde{\mu} = \begin{bmatrix}\mu\\ \mu'\\ \mu''\end{bmatrix} \approx \begin{bmatrix}\eta\\ 0\\ 0\end{bmatrix}$$

Under this assumption, the precision-weighted prediction error ξ'_z approximates $2\Gamma_z\lambda_z^2 y'_{h,i}$, and thus signals whether neighbors are instantaneously approaching or moving away from the focal agent, where $\xi'_z < 0$ indicates they are approaching and $\xi'_z > 0$ indicates they are moving away. This in turn determines whether the update to the focal agent's action $\mathbf{v}_i$ is repulsive or attractive, as its sign determines the direction of the social force vector $\Delta\hat{\mathbf{r}}$. Although the first order distance $y_{h,i}$ does not directly drive action, it does so indirectly through its effect on inference of μ'. If we consider the case when the sensed distance $y_{h,i}$ drops below the setpoint η, then one can reason through the cascade of prediction errors that ultimately lead to a repulsive force. As a direct consequence of a drop in $y_{h,i}$ below μ, sensory prediction errors ξ_z will become negative, whose

minimization will require μ to move below η. This process in turn incurs slower-moving (negative) model prediction errors ξ_ω, whose minimization drives μ back to its fixed point of η, given the dynamic constraint for the beliefs to relax to their fixed point. In order to accomplish this upward movement of μ, either the rate of change of μ or the sensed distance itself must be positive, i.e., $\dot{\mu} > 0$ or $y_{h,i} > 0$. In the absence of positive $y_{h,i}$, model prediction errors will drive μ' (and hence $\dot{\mu}$) above 0. This temporarily sets a larger radius of repulsion, i.e., a larger range of $y'_{h,i}$ for which ξ'_z is negative and for which repulsive forces impact the focal agent's velocity. This causes the agent to move away from its neighbors and thus further increase $y_{h,i}$, under the assumption that the agent's prediction of the distance dynamics are correlated with the true change in $x_{h,i}$. Belief updating and action thus work together to accelerate the return of μ towards η and $\tilde{\xi}_z, \tilde{\xi}_\omega$ towards 0; for this reason active inference is often described as an account of action and perception driven by 'self-fulfilling prophecy' (6).

In order to imbue action with a more direct coupling to the neighbors' distances as is done in the classical self-propelled particle models, rather than the velocity of the distance, one could hand-craft the sensorimotor contingency term $\nabla_{\mathbf{v}_i}\tilde{y}_{h,i}$ to enforce a coupling between $y_{h,i}$ and $\mathbf{v}_i$. This would render the action rule equivalent to a 'soft'-form of PD control (14), where errors on both the first order state $(y_{h,i} - \mu) \approx (y_{h,i} - \eta)$ and its derivative $(y'_{h,i} - \mu') \approx y'_{h,i}$ would drive changes to the velocity.

Extending to multiple sensory sectors. The results of the previous sections can be straightforwardly extended to the multivariate case as explored in the main text. The focal agent now senses the local distance computed across a set of distinct sensory sectors. For the model explored in the current work, we split up the computation of the local distance variable into a set of L sensory adjacent sectors that comprise an arc of a given angle, relative to the agent's heading vector $\mathbf{v}_i$. We define the multivariate distance hidden state as follows (dropping the focal agent index i from the sector-specific hidden states to avoid subscript overload):

$$\mathbf{x}_{h,i} = \begin{bmatrix} x_{h,1} \\ x_{h,2} \\ \vdots \\ x_{h,L} \end{bmatrix} \qquad [47]$$

$$\text{where} \quad x_{h,l} \triangleq \frac{1}{K_l} \sum_{j \in N_l} \|\Delta \mathbf{r}_{ij}\|$$

where N_l is the set of neighbors in the lth sensory sector, and $K_l = |N_l|$ (c.f., Eq. (39)). As with the scalar hidden state defined above, we also equip the vector of sector distances $\mathbf{x}_{h,i}$ with corresponding sector-specific, generalized observations $\tilde{\mathbf{y}}_{h,i}$, i.e.

$$\mathbf{y}_{h,i} = \mathbf{x}_{h,i} + \mathbf{z}_{h,i} \qquad [48]$$
$$\mathbf{y}'_{h,i} = \mathbf{x}'_{h,i} + \mathbf{z}'_{h,i}$$
$$\text{where} \quad p(\tilde{\mathbf{z}}_{h,i}) = \mathcal{N}(\tilde{\mathbf{z}}_{h,i}; \mathbf{0}, \tilde{\Sigma}_{\mathbf{z},h}) \qquad [49]$$

such that the focal individual now observes a vector of local (noise-perturbed) distances and their first orders of motion. Note that the generalized covariance matrix here $\tilde{\Sigma}_{\mathbf{z},h}$ is now a $2L \times 2L$ size matrix, that encodes the covariance structure between sector-specific noise and their generalized orders. For all simulations we generated uncorrelated noise across the different sectors, although spatially-smooth noise could be modelled by introducing off diagonal elements in $\tilde{\Sigma}_{\mathbf{z},h}$, i.e., $\mathbb{E}[z_{h,l} z_{h,k}] \neq 0$.

The agent's generative model is also extended to the multivariate state-space formulation we began with, using a vector of generalised hidden states $\tilde{\mathbf{x}} = (\tilde{x}_1, \tilde{x}_2, ..., \tilde{x}_L)$ to estimate the local distance within each sensory sector. Belief-updating consists in updating a vector of generalised means $\tilde{\boldsymbol{\mu}}$ through integration of Eq. (37).

The action update has an identical form as before, except now the sensorimotor contingency term $\nabla_{\mathbf{v}_i}\tilde{\mathbf{y}}_{h,i}(\mathbf{v_i})$ is a collection of partial derivative vectors, one for each sensory sector:

$$\frac{d\mathbf{v}_i}{dt} = -\nabla_{\mathbf{v}_i}\tilde{\mathbf{y}}_{h,i}(\mathbf{v}_i)^\top \nabla_{\tilde{\mathbf{y}}_{h,i}} F_L$$

$$\nabla_{\mathbf{v}_i}\tilde{\mathbf{y}}_{h,i}(\mathbf{v_i}) = \begin{bmatrix} \nabla_{\mathbf{v}_i}\mathbf{y}_{h,i}(\mathbf{v}_i) \\ \nabla_{\mathbf{v}_i}\mathbf{y}'_{h,i}(\mathbf{v}_i) \end{bmatrix} = \begin{bmatrix} \mathbf{0} \\ \vdots \\ \mathbf{0} \\ \nabla_{\mathbf{r}_i} x_{h,1} \\ \nabla_{\mathbf{r}_i} x_{h,2} \\ \vdots \\ \nabla_{\mathbf{r}_i} x_{h,L} \end{bmatrix} \qquad [50]$$

The last L rows of this Jacobian matrix encode the gradients of the sector-specific distance velocities $y'_{h,l}$ with respect to the focal agent's action; these partial derivatives are vectors pointing from the average position of the neighbors in sector l towards the focal individual. When we combine the Jacobian matrix in Eq. (50) with the sensory prediction error term $\tilde{\mathbf{y}}_{h,i}$ (i.e., the free energy gradients $\nabla_{\tilde{\mathbf{y}}_{h,i}} F_L$), we are left with the following update for the velocity:

$$\begin{aligned} \frac{d\mathbf{v}_i}{dt} &= \boldsymbol{\xi}'_z \cdot \Delta\hat{\mathbf{R}} = \begin{bmatrix} \xi'_{z,1} & \xi'_{z,2} & \dots & \xi'_{z,L} \end{bmatrix} \cdot \begin{bmatrix} \Delta\hat{\mathbf{r}}_1 \\ \Delta\hat{\mathbf{r}}_2 \\ \vdots \\ \Delta\hat{\mathbf{r}}_L \end{bmatrix} \\ &= \sum_{l=1}^{L} \xi'_{z,l} \Delta\hat{\mathbf{r}}_l = 2\Gamma_z \lambda_z^2 \sum_{l=1}^{L} (y'_{h,l} - \mu'_l) \Delta\hat{\mathbf{r}}_l \qquad [51] \\ \text{where} \quad \Delta\hat{\mathbf{r}}_l &= \frac{1}{K_l} \sum_{j \in N_l} \frac{\Delta\mathbf{r}_{ij}}{||\Delta\mathbf{r}_{ij}||} \end{aligned}$$

The action thus becomes a weighted sum of 'sector-vectors' $\Delta\hat{\mathbf{r}}_l$, which are vectors pointing from the focal agent's position $\mathbf{r}_i$ towards the average position of the neighbors in N_l. The weights that scale each $\Delta\hat{\mathbf{r}}_l$ are the precision-weighted prediction errors associated with velocity observations emanating from the appropriate sector $\xi'_{z,l} \propto (y'_{h,l} - \mu'_l)$. The fact we can pull the spatiotemporal precision terms $2\Gamma_z\lambda_z^2$ outside the sum over sector-vectors, inherits from a between-sector independence assumption, built into the agent's sensory likelihood model $P(\tilde{\mathbf{y}}|\tilde{\mathbf{x}})$ (see Eq. (31)). If the generative model allowed for between-sector correlations (i.e. $\Sigma^{\mathbf{z}}$ was not diagonal), then the action update would include cross-terms that couple prediction errors from one sector to the sector-vector from another sector.

An active inference agent equipped with such a multivariate representation of the local neighbor-distances thus engages in a sort of 'predictive balancing-act', differentially responding more or less to each part of its sensory field in accordance with how much sensations deviate from their posterior expectations μ'_l, where the sign and degree of this deviation is scored by $\xi'_{z,l}$.

Gaussianity of the generative model. One may question the use of a Gaussian form for the generalized noises $\tilde{\mathbf{z}}, \tilde{\boldsymbol{\omega}}$ and a linear form for $\tilde{\mathbf{f}}$ and $\tilde{\mathbf{g}}$. These assumptions guarantee a simple generative model whose free energy gradients (with respect to both the state belief $\tilde{\boldsymbol{\mu}}$ and action $\mathbf{v}$) are linear in the generalized distance observations $\tilde{\mathbf{y}}$. The reason we use a Gaussian form here, is to achieve those simple, linear free energy gradients, i.e., those which align with the classical social forces seen in SPP models (more specifically, the selective attraction-and-repulsion forces first described in (19)); in other words, it is exactly an active inference model equipped with this sort of model (and observations with two generalized coordinates, aka position and velocity) that acts in a way equivalent to being driven by vectorial social forces. Note that this also means the Laplace approximation to the posterior over $\tilde{\mathbf{x}}$ is not an approximation at all, but leads to exact inference.

Existing social force models where the forces are linear in the state, can thus be read as special cases of active inference agents equipped with Gaussian generative models – this is a close cousin to the relationship between linear PID controllers and Gaussian active inference models derived in (14). However, if we were to use a different, non-Gaussian of the generative model (either through breaking Gaussianity of the noise model or by introducing nonlinear forms of $\tilde{\mathbf{f}}, \tilde{\mathbf{g}}$), then the updates to the beliefs and actions would no longer be linear functions of the generalized posterior means $\tilde{\boldsymbol{\mu}}$ and observations $\tilde{\mathbf{y}}$, but could be arbitrary, nonlinear functions of these variables – and the resulting social forces would have no guaranteed interpretation in terms of attraction and repulsion. Such extensions are an interesting avenue for future work; one interesting possibility would be to explore new extensions of social forces, where the noises $\tilde{\mathbf{z}}, \tilde{\boldsymbol{\omega}}$ are assumed to belong to the exponential family and that the dynamics model $\tilde{\mathbf{f}}$ is smooth and contains attracting fixed points (i.e., regions of $\tilde{\mathbf{x}}$ where the derivatives of $\tilde{\mathbf{f}}$ vanish). A obvious consequence of such assumptions, is that when the posterior belief $\tilde{\boldsymbol{\mu}}$ is near these fixed points, i.e., $\tilde{\boldsymbol{\mu}} \approx \arg\min_{\tilde{\mathbf{x}}} \nabla_{\tilde{\mathbf{x}}} \tilde{\mathbf{f}}(\tilde{\mathbf{x}}) \implies \tilde{\boldsymbol{\mu}}' = 0$, we would still recover attractive and repulsive social forces whose magnitude would be approximately linear in the distance observations $\tilde{\mathbf{y}}$. This is equivalent to making a locally-quadratic (i.e., Laplace) approximation to the free energy landscape around its minima – this corresponds to just those points in belief-space where the posterior is well-approximated by a Gaussian.

2. Alignment forces from active inference on angles

In previous sections we have shown how repulsive and attractive forces emerge from active inference models in which the agent entertains a latent representation of the average local distance between itself and its neighbors, and how its heading direction couples to (the derivative of) that variable. In this section we derive alignment-based social forces, like those that appear in the Reynolds, Couzin, and Vicsek models (15, 17, 18), as a special case of active inference, where an agent infers the (cosine) angle between its own heading and that of its neighbors, and acts under the prior belief that this angle tends to 0.

As before, we start with a generative model that represents a generalised latent variable $\tilde{x}_\phi$ that evolves in time with Gaussian additive fluctuations $\tilde{\omega}_\phi$. We use the ϕ subscript to distinguish this angle-tracking latent variable from the distance-tracking variable of the previous section. We truncate the generalized representation of this state at second order, i.e. $\tilde{x}_\phi = \{x_\phi, x'_\phi\}$, leading to a dynamical equation and corresponding likelihood of the following form:

$$\dot{x}_\phi = -\alpha_\phi(x_\phi - 1) + \omega_\phi$$
$$\dot{x}'_\phi = -\alpha_\phi x'_\phi + \omega'_\phi$$
$$\implies p(D\tilde{x}_\phi|\tilde{x}_\phi) = \mathcal{N}(D\tilde{x}_\phi; \tilde{f}_\phi, \tilde{\Sigma}_{\omega_\phi}) \quad [52]$$
$$\text{where} \quad \tilde{f}_\phi = \begin{bmatrix} -\alpha_\phi(x_\phi - 1) \\ -\alpha_\phi x'_\phi \end{bmatrix}, \quad \tilde{\Sigma}_{\omega_\phi} = \begin{bmatrix} \sigma^2_{\omega_\phi} & 0 \\ 0 & \sigma^2_{\omega'_\phi} \end{bmatrix} \quad [53]$$

The observation model describes a mapping from the 0th-order state to a corresponding observation thereof, perturbed again by Gaussian innovations:

$$y_\phi = x_\phi + z_\phi$$
$$\implies p(y_\phi|x_\phi) = \mathcal{N}(y_\phi; x_\phi, \sigma^2_{z_\phi}) \quad [54]$$

Following the same steps as we did previously for the multivariate, distance-tracking generative model, we can write down the Laplace-approximated variational free energy of this model as a quadratic function of the observations and generalized means $\tilde{\mu}_\phi$:

$$F_L \propto \varepsilon_{z_\phi}^\top \Pi_{z_\phi} \varepsilon_{z_\phi} + \tilde{\varepsilon}_{\omega_\phi}^\top \tilde{\Pi}_{\omega_\phi} \tilde{\varepsilon}_{\omega_\phi}$$
$$\text{where} \quad \varepsilon_{z_\phi} \triangleq y_\phi - \mu_\phi$$
$$\tilde{\varepsilon}_{\omega_\phi} \triangleq D\tilde{\mu}_\phi - \tilde{f}_\phi$$

The agent performs a gradient descent on F_L to infer the value of $\tilde{x}_\phi$ in light of sensory observations. This inference is encoded by a Gaussian variational posterior with mean $\tilde{\mu}_\phi$. As before, we can tune model parameters such that inference is strongly biased by the dynamics model $\tilde{f}_\phi$, where the zeroth-order of motion $\mu_\phi \approx 1$. The reason we set the set-point at 1 becomes evident when we consider the generation of sensory data and actions.

Assume that the focal agent with index i observes the local average cosine angle between its own heading vector $\mathbf{v}_i$ and those of its neighbors $\mathbf{v}_j, j \in N_{in}$, where neighbors are once again defined by membership in some interaction zone[‡]:

$$y_\phi = \frac{1}{K} \sum_{j \in N_{in}} \mathbf{v}_i^\top \mathbf{v}_j = \langle \cos(\theta_{ij}) \rangle_{N_{in}} \quad [55]$$

where the equivalence between the dot products and the cosine angle is assured when we assume all $\mathbf{v}_k, k \in \{i\} \cup N_{in}$ have unit magnitude. Recall that if two unit-magnitude vectors $\mathbf{v}_i, \mathbf{v}_j$ are parallel, their dot product (cosine angle) is 1. When we once again assume that agents act by adjusting their heading direction, then the action update given the continuous active inference rule in Eq. (22) has the following form:

$$\frac{d\mathbf{v}_i}{dt} = -\frac{1}{\sigma^2_{z_\phi}}(y_\phi - \mu_\phi)\hat{\mathbf{v}} \approx (1 - y_\phi)\hat{\mathbf{v}} \quad [56]$$
$$\text{where} \quad \hat{\mathbf{v}} = \frac{1}{K} \sum_{j \in N_{in}} \mathbf{v}_j \quad [57]$$

The approximation in the first line holds when we assume the sensory variance $\sigma^2_{z_\phi}$ is 1 and the dynamics prior (either via increasing α or decreasing $\sigma^2_{\omega_\phi}$) dominates inference such that $\mu_\phi \approx 1$. In this case, the focal agent i then updates its velocity using the average neighbor velocity. This is proportional to the alignment force in e.g. (15, 18), except that it is also scaled by how unaligned the focal individual is with its neighbourhood, scored by $1 - y_\phi$.

3. Online parameter estimation

In this section we derive update rules for the generative model parameters using a simple gradient descent scheme on the Laplace-approximated variational free energy. In the active inference literature this process of updating parameters, as opposed to beliefs about states, is often analogized to online learning or neural plasticity (20, 21).

‡ For notational convenience and because it doesn't change the derivations, we omit observation noise on y_ϕ.

Updating sensory smoothness. In this section we derive an update equation for the sensory smoothness parameter λ_z, which captures the generative model's assumptions about the temporal autocorrelation structure of sensory noise z.

Recall the formulation of state space models in generalized coordinates of motion in Section 1. In addition to providing a concise description of local paths of the state $\vec{x}_t$ in terms of its higher derivatives $x', x'', ..., x^{[n]}$, stochastic differential equations in generalized coordinates also allow one to express *serial correlations* in the noises at the first order z, by assuming that it can be differentiated (has non-zero, smooth autocovariance) and represented in terms of hierarchical or generalized noises $z', z'', z''', ..., z^{[n]}$.

Recall the parameterization of the generalized sensory precision $\tilde{\Pi}^z$ as a factorization into two precision matrices, that respectively represent agent's beliefs about the 'spatial' and 'temporal' covariance structure. We parameterize these with the two precision parameters Γ_z and λ_z. Γ_z encodes the agent's belief about the overall magnitude of the fluctuations, and λ_z encodes beliefs about their their serial correlations in time, assuming a Gaussian form for their autocorrelation:

$$
\begin{aligned}
\tilde{\Pi}^z &= S(\lambda_z) \otimes \Pi(\Gamma_z) \\
\Pi(\Gamma_z) &= \begin{bmatrix} \Gamma_{11} & & & \\ & \Gamma_{22} & & \\ & & \ddots & \\ & & & \Gamma_{LL} \end{bmatrix} \\
S(\lambda_z) &= \begin{bmatrix} 1 & 0 & -\frac{1}{2\lambda_z^2} & \cdots \\ 0 & \frac{1}{2\lambda_z^2} & 0 & \\ -\frac{1}{2\lambda_z^2} & 0 & \frac{3}{4\lambda_z^4} & \\ \vdots & & & \ddots \end{bmatrix}^{-1}
\end{aligned} \qquad [58]
$$

We implement a form of behavioral plasticity by allowing agents to update λ_z using observations. We accomplish this using a gradient descent on variational free energy:

$$
\frac{d\lambda_z}{dt} = -\kappa_\theta \frac{\partial F}{\partial \lambda_z} \qquad [59]
$$

where the 'learning rate' κ_θ is typically set to be at least an order of magnitude lower than the update rate of inference κ_μ; in all simulations we use $\kappa_\theta = 0.001$ and $n_{\text{LearnIter}} = 1$ iteration. This enforces a separation of timescales that is typical in generalized filtering and state-space models that perform simultaneous state- and parameter-estimation (3, 5, 14).

To compute the gradients of the variational free energy with respect to λ_z, we can start by expressing those components of the (Laplace-approximated) variational free energy that depend on λ_z:

$$
F(\lambda_z) = \tilde{\varepsilon}_{\mathbf{z}}^\top \tilde{\Pi}^{\mathbf{z}} \tilde{\varepsilon}_{\mathbf{z}} - \ln\left(\det \tilde{\Pi}^{\mathbf{z}}\right) \qquad [60]
$$

where we only have included the terms that depend on the sensory precision $\tilde{\Pi}^z$ due to its dependence on λ_z. The full gradient is then simply:

$$
\frac{\partial F}{\partial \lambda_z} = \frac{\tilde{\varepsilon}_{\mathbf{z}}^\top \tilde{\Pi}^{\mathbf{z}} \tilde{\varepsilon}_{\mathbf{z}}}{\partial \lambda_z} - \frac{\partial \ln\left(\det \tilde{\Pi}^{\mathbf{z}}\right)}{\partial \lambda_z} \qquad [61]
$$

Starting with the case of a single sensory sector $L = 1$, then the generalized prediction error $\tilde{\varepsilon}_{\mathbf{z}}$ is a vector of prediction errors, one for each order of motion: $\tilde{\varepsilon}_z = \{\varepsilon_z, \varepsilon_z', \varepsilon_z'', ...\}$ where a sensory prediction error at a given order of motion is simply: $\varepsilon_z^{[n]} = y^{[n]} - \tilde{g}^{[n]}$, where the n subscript refers to an order of differentiation. In the case of 3 generalized coordinates for the simple scalar case:

$$
\begin{aligned}
\frac{\partial F}{\partial \lambda_z} &= 4\Gamma_z\lambda_z(\varepsilon_z')^2 + \varepsilon_z''(8\Gamma_z\varepsilon_z''\lambda_z^3 + 2\Gamma_z\varepsilon_z\lambda_z) + 2\Gamma_z\lambda_z\varepsilon_z\varepsilon_z'' - \frac{6}{\lambda_z} \\
&= 4\Gamma_z\lambda_z(\varepsilon_z\varepsilon_z'' + (\varepsilon_z')^2 + 2\lambda_z^2(\varepsilon_z'')^2) - \frac{6}{\lambda_z}
\end{aligned} \qquad [62]
$$

Meaning that the update for the λ_z parameter can be simplified to (omitting the learning rate κ_θ):

$$
\frac{d\lambda_z}{dt} = -4\Gamma_z\lambda_z(\varepsilon_z\varepsilon_z'' + (\varepsilon_z')^2 + 2\lambda_z^2(\varepsilon_z'')^2) + \frac{6}{\lambda_z} \qquad [63]
$$

In the case of the distance-tracking generative model we explore in the main text, we assume that the agents can only observe the 0th (position, y) and 1st (velocity, y') orders of motion of the hidden states $\tilde{x}$. This means there are no longer 2nd-order prediction errors ε_z'' and the update becomes even simpler:

$$\begin{aligned}\frac{d\lambda_z}{dt} &= -4\Gamma_z\lambda_z(\varepsilon_z')^2 + \frac{6}{\lambda_z} \\ &\approx -4\Gamma_z\lambda_z(y_{h,i}')^2 + \frac{6}{\lambda_z}\end{aligned} \tag{64}$$

where approximation in the second line results in the case of 'biased' inference, i.e., $\mu \approx \eta \implies \mu' \approx 0$, allowing us to replace the velocity prediction error $y_{h,i}' - \mu'$ with $y_{h,i}'$.

Given that spatial and temporal precisions are independent from each other due to the factorization of the generalized precision matrix, and further given the diagonal structure of the spatial precision $\Pi_{\mathbf{z}}$ (i.e., independence in random fluctuations across sensory sectors), we can write an update for λ_z that is a sum of squared prediction errors across sensory sectors:

$$\frac{d\lambda_z}{dt} \approx -4\Gamma_z\lambda_z\sum_{l=1}^{L}(y_{h,l}')^2 - \frac{6L}{\lambda_z} \tag{65}$$

The quadratic form of this update means that the update to the smoothness parameter decays in proportion with the overall magnitude of the velocity prediction errors, regardless of its sign. This means that if the distance is fluctuating quickly in any direction, then the agent will infer that fluctuations are slightly-less serially-correlated at the 0th order, reflected by a decrease in λ_z.

4. Adding a target representation into the generative model

As described in the main text, it is straightforward to add an additional observation model and dynamics model to an agent's generative model to represent the distance between itself and some abstract spatial target, which in the context of the collective information transfer experiments, we represent with $\mathbf{T}$:

$$\begin{aligned}\dot{x}_{\text{target}} &= -\alpha_{\text{target}}x_{\text{target}} + \omega_{\text{target}} & y_{\text{target}} &= x_{\text{target}} + z_{\text{target}} \\ \dot{x}_{\text{target}}' &= -\alpha_{\text{target}}x_{\text{target}}' + \omega_{\text{target}}' & y_{\text{target}}' &= x_{\text{target}}' + z_{\text{target}}'\end{aligned} \tag{66}$$

We truncate the generalized hidden states at third order $\tilde{x}_{\text{target}} = (x_{\text{target}}, x_{\text{target}}', x_{\text{target}}'')$ and the observations at second order $\tilde{y}_{\text{target}} = (y_{\text{target}}, y_{\text{target}}')$. When the agent assumes the generalized noises $\tilde{\omega}_{\text{target}}$ and $\tilde{z}_{\text{target}}$ are zero-mean and normally-distributed with covariances $\tilde{\Sigma}^{\omega_{\text{target}}}$ and $\tilde{\Sigma}^{z_{\text{target}}}$ and leverage the Laplace approximation exactly as we did in the previous section, then we can supplement the Laplace-approximated free energy in Eq. (35) with additional terms corresponding to target-related prediction errors:

$$F_L \propto \frac{1}{2}\left[\tilde{\boldsymbol{\varepsilon}}_{z\text{-Soc}}^\top\tilde{\Pi}^{\boldsymbol{z}\text{-Soc}}\tilde{\boldsymbol{\varepsilon}}_{z\text{-Soc}} + \tilde{\boldsymbol{\varepsilon}}_{\omega\text{-Soc}}^\top\tilde{\Pi}^{\boldsymbol{\omega}\text{-Soc}}\tilde{\boldsymbol{\varepsilon}}_{\omega\text{-Soc}} + \tilde{\varepsilon}_{z\text{-Tar}}^\top\tilde{\Pi}^{z\text{-Tar}}\tilde{\varepsilon}_{z\text{-Tar}} + \tilde{\varepsilon}_{\omega\text{-Tar}}^\top\tilde{\Pi}^{\omega\text{-Tar}}\tilde{\varepsilon}_{\omega\text{-Tar}}\right] + C \tag{67}$$

Here we use the suffixes "-Soc" or "-Tar" to indicate 'social' relevant information (related to the average neighbor distance) and the 'target' prediction errors. C captures all the additional terms (log determinants of precision matrices, etc.) that are constant with respect to the posterior means $\tilde{\boldsymbol{\mu}} = (\tilde{\boldsymbol{\mu}}_{\text{Social}}, \tilde{\mu}_{\text{Target}})$. Following the same reasoning as used to derive the inference and action rules for the case of the social distance hidden states and observations($\tilde{\mathbf{x}}_{\text{Social}}, \tilde{\mathbf{y}}_{\text{Social}}$), we can do the same to derive active inference rules for the target-relevant hidden states and observations $\tilde{x}_{\text{target}}, \tilde{y}_{\text{target}}$:

$$\begin{aligned}\frac{d\tilde{\boldsymbol{\mu}}_{\text{Social}}}{dt} &= D\tilde{\boldsymbol{\mu}}_{\text{Social}} - \nabla_{\tilde{\boldsymbol{\mu}}_{\text{Social}}}F_L(\tilde{\boldsymbol{\mu}}_{\text{Social}}, \tilde{\mathbf{y}}_{\text{Social}}) & \frac{d\mathbf{v}}{dt} &= -\left(\nabla_{\mathbf{v}}F_L(\tilde{\boldsymbol{\mu}}_{\text{Social}}, \tilde{\mathbf{y}}_{\text{Social}}) + \nabla_{\mathbf{v}}F_L(\tilde{\mu}_{\text{Target}}, \tilde{y}_{\text{Target}})\right) \\ \frac{d\tilde{\mu}_{\text{Target}}}{dt} &= D\tilde{\mu}_{\text{Target}} - \nabla_{\tilde{\mu}_{\text{Target}}}F_L(\tilde{\mu}_{\text{Target}}, \tilde{y}_{\text{Target}})\end{aligned} \tag{68}$$

Where expanding the free energy gradients on the right equation leads to an expression for the action update in terms of a precision-weighted sum of vectors, appearing in Eq. (14) in the main text.

5. Numerical methods

We used a forwards Euler-Maruyama scheme to the integrate a (Itô-style) stochastic differential equation for the positions of all agents over time:

$$d\mathbf{r}_t = \mathbf{v}_t dt + \sigma_a dW_t \qquad [69]$$

where the variance of 'action noise' σ_a^2 was set to 0.01 for all experiments unless explicitly stated otherwise. We used a step size of $\Delta t = 0.01s$ in the integration. For the current timestep τ in 'simulation time', we used a simple forwards Euler scheme to integrate the differential equations used for belief updating (see Eq. (37)) and action (see Eq. (43)) for each agent in parallel. We use the positions and heading vectors of all agents from the previous integration timestep ($\tau - \Delta t$) to generate the observations for the current timestep.

The collective information transfer experiments were performed using custom Julia code, and all other simulations were implemented in JAX using custom code. To accelerate the parameter scans over p_{inf}, $\Gamma_{z\text{-Social}}$, and $\Gamma_{z\text{-Target}}$ to create the results in Figure 3 in the main text, we used the high-performance computing clusters (Cobra and Draco) provided by the Max Planck Computing and Data Facility.

Table S1. Default parameter configuration used in numerical simulations (unless otherwise stated).

Parameter	Value	Type
Δt (Euler integration step, in seconds)	0.01	generative process
Number of sensory sectors	4	generative process
Sector angle (in degrees $^\circ$)	60	generative process
R_0 (interaction radius, in arbitrary units)	5	generative process
σ_a^2	0.01	generative process
$\sigma_{z,h}^2$	0.01	generative process
$\sigma_{z',h}^2$	0.01	generative process
Γ_z	1.0	generative model
Γ_ω	1.0	generative model
λ_z	1.0	generative model
λ_ω	1.0	generative model
α	0.5	generative model
α_{target}	0.5	generative model
η	1.0	generative model
Number of generalised coordinates (x)	3	hyperparameter
Number of generalised coordinates (y)	2	hyperparameter
κ_μ	0.1	hyperparameter
$n_{\text{InferIter}}$	1	hyperparameter
κ_a	0.1	hyperparameter
$n_{\text{ActionIter}}$	1	hyperparameter
κ_θ	0.001	hyperparameter
$n_{\text{LearnIter}}$	1	hyperparameter

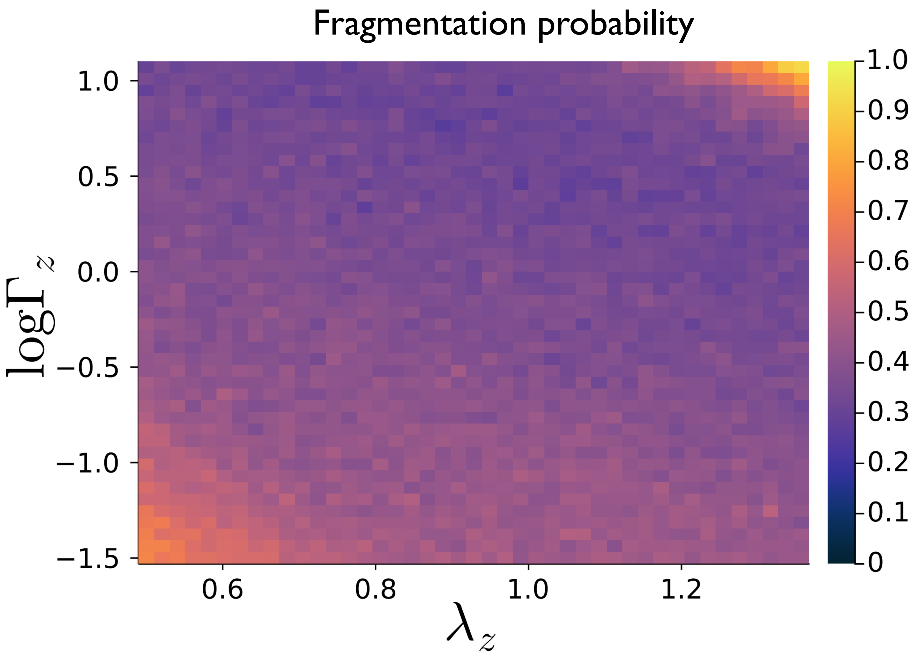

Fig. S1. Fragmentation probability as a function of the two precision parameters $\log \Gamma_z$ and λ_z. Fragmentation probability was quantified as the proportion of trials (out of 500 independent trials per condition) where the group fragmented. A trial was considered fragmented if least one individual was further than 2.0 dimensionless units from all other individuals for at least 3 of the last 10 seconds of the 15-second trial. All other parameters are identical to those used in Figure 2B in the main text.

Movie S1. Example of a simulation of $N = 96$ agents that includes a dynamic transition from polarized to milling regime. Parameters: $\sigma^2_{z',h} = 0.05$; Sector angle $= 80°$; $R_0 = 10$ units; $\kappa_a = 0.2$; $\lambda_\omega = 0.5$; $\lambda_z = 2.0$. Unless specified, all remaining parameters are as listed in Table S1.

Movie S2. Example of a polarized group of $N = 64$ agents. Parameters: Sector angle $= 80°$;; $\kappa_a = 0.2$; $\lambda_\omega = 0.5$; $\lambda_z = 1.5$.

Movie S3. Example of a milling regime observed in $N = 64$ agents. Parameters: $\sigma^2_{z',h} = 0.04$; Sector angle $= 80°$; $\alpha = 1.0$; $\kappa_a = 0.2$; $\lambda_\omega = 0.8$; $\lambda_z = 1.8$.

Movie S4. Example of a disordered regime observed in $N = 96$ agents. Parameters: Number of sensory sectors $= 2$; Sector angle $= 160°$; $R_0 = 10.0$ units; $\alpha = 0.2$; $\eta = 0.5$, $\kappa_a = 0.2$; $\lambda_\omega = 0.1$; $\lambda_z = 1.787$.

Movie S5. Metastable 'snaking' configuration observed in $N = 64$ agents. Parameters: $\sigma^2_{z',h} = 0.04$; Sector angle $= 80°$; $\alpha = 0.1$; $\kappa_a = 0.2$; $\lambda_\omega = 0.5$; $\lambda_z = 2.2$.

References

1. K Friston, K Stephan, B Li, J Daunizeau, Generalised filtering. *Math. Probl. Eng.* **2010** (2010).
2. K Friston, Hierarchical models in the brain. *PLoS computational biology* **4**, e1000211 (2008).
3. B Balaji, K Friston, Bayesian state estimation using generalized coordinates. *Signal Process. Sens. Fusion, Target Recognit. XX* **8050**, 716–727 (2011).
4. KJ Friston, Variational filtering. *NeuroImage* **41**, 747–766 (2008).
5. KJ Friston, N Trujillo-Barreto, J Daunizeau, Dem: a variational treatment of dynamic systems. *Neuroimage* **41**, 849–885 (2008).
6. CL Buckley, CS Kim, S McGregor, AK Seth, The free energy principle for action and perception: A mathematical review. *J. Math. Psychol.* **81**, 55–79 (2017).
7. K Friston, et al., The free energy principle made simpler but not too simple. *Phys. Reports* **1024**, 1–29 (2023).
8. K Friston, A theory of cortical responses. *Philos. transactions Royal Soc. B: Biol. sciences* **360**, 815–836 (2005).
9. K Friston, S Kiebel, Predictive coding under the free-energy principle. *Philos. transactions Royal Soc. B: Biol. sciences* **364**, 1211–1221 (2009).
10. Y Huang, RP Rao, Predictive coding. *Wiley Interdiscip. Rev. Cogn. Sci.* **2**, 580–593 (2011).
11. RA Adams, S Shipp, KJ Friston, Predictions not commands: active inference in the motor system. *Brain Struct. Funct.* **218**, 611–643 (2013).
12. K Friston, What is optimal about motor control? *Neuron* **72**, 488–498 (2011).
13. T Parr, G Pezzulo, KJ Friston, *Active inference: the free energy principle in mind, brain, and behavior.* (MIT Press), (2022).
14. M Baltieri, CL Buckley, Pid control as a process of active inference with linear generative models. *Entropy* **21**, 257 (2019).
15. ID Couzin, J Krause, R James, GD Ruxton, NR Franks, Collective memory and spatial sorting in animal groups. *J. theoretical biology* **218**, 1–11 (2002).
16. I AOKI, A simulation study on the schooling mechanism in fish. *NIPPON SUISAN GAKKAISHI* **48**, 1081–1088 (1982).
17. CW Reynolds, Flocks, herds and schools: A distributed behavioral model in *Proceedings of the 14th annual conference on Computer graphics and interactive techniques.* pp. 25–34 (1987).
18. T Vicsek, A Czirók, E Ben-Jacob, I Cohen, O Shochet, Novel type of phase transition in a system of self-driven particles. *Phys. review letters* **75**, 1226 (1995).
19. P Romanczuk, ID Couzin, L Schimansky-Geier, Collective motion due to individual escape and pursuit response. *Phys. Rev. Lett.* **102**, 010602 (2009).
20. K Friston, et al., Active inference and learning. *Neurosci. & Biobehav. Rev.* **68**, 862–879 (2016).
21. L Da Costa, et al., Active inference on discrete state-spaces: A synthesis. *J. Math. Psychol.* **99**, 102447 (2020).
